# Supplementary material for: Study protocol: computerised cognitive testing in a cohort of people with frontotemporal dementia
Source: BMJ Open. 2022 Aug 5;12(8):e055211. doi: 10.1136/bmjopen-2021-055211 (PMC9362783; doi:10.1136/bmjopen-2021-055211)
Supplement: Author's manuscript [file bmjopen-2021-055211.draft_revisions.pdf]

BMJ Open

BMJ Open is committed to open peer review. As part of this commitment we make the peer review history of every article we publish publicly available.

When an article is published we post the peer reviewers' comments and the authors' responses online. We also post the versions of the paper that were used during peer review. These are the versions that the peer review comments apply to.

The versions of the paper that follow are the versions that were submitted during the peer review process. They are not the versions of record or the final published versions. They should not be cited or distributed as the published version of this manuscript.

BMJ Open is an open access journal and the full, final, typeset and author-corrected version of record of the manuscript is available on our site with no access controls, subscription charges or pay-per-view fees (http://bmjopen.bmj.com).

If you have any questions on BMJ Open's open peer review process please email info.bmjopen@bmj.com

BMJ Open

Ignite – a novel computerized cognitive assessment battery for frontotemporal dementia

Journal:	*BMJ Open*
Manuscript ID	bmjopen-2021-055211
Article Type:	Protocol
Date Submitted by the Author:	08-Aug-2021
Complete List of Authors:	Moore, Katrina M; UCL Queen Square Institute of Neurology, Department of Neurodegenerative Disease<br>Convery, Rhian S.; UCL Queen Square Institute of Neurology, Department of Neurodegenerative Disease<br>Rohrer, Jonathan; UCL Queen Square Institute of Neurology, Department of Neurodegenerative Disease
Keywords:	Dementia < NEUROLOGY, Adult neurology < NEUROLOGY, Delirium & cognitive disorders < PSYCHIATRY

SCHOLARONE™
Manuscripts

STUDY PROTOCOL

Main title

Ignite – a novel computerized cognitive assessment battery for frontotemporal dementia

Authors

Katrina M Moore PhD[1], Rhian S. Convery MSc[1], Jonathan D Rohrer FRCP PhD[1]

Affiliations

[1]Dementia Research Centre, Department of Neurodegenerative Disease, UCL Queen Square Institute of Neurology, London, UK.

Corresponding author

Dr Jonathan Rohrer, Dementia Research Centre, Department of Neurodegenerative Disease, UCL Institute of Neurology, Queen Square, London, WC1N 3BG, j.rohrer@ucl.ac.uk

For peer review only

Abstract

Introduction: The term frontotemporal dementia (FTD) refers to a heterogeneous group of neurodegenerative disorders affecting the frontal and temporal lobes. Cognitively, impairment of executive function and social cognition predominate across the FTD spectrum although other domains can be affected. Traditionally, cognition is tested through standard 'pen and paper' tasks in FTD. However, recent attempts have been made across other neurodegenerative disorders such as Alzheimer's disease to develop computerized batteries that allow more accurate and sensitive detection of cognitive impairment.

Methods: In this paper we describe the development of Ignite, a novel battery of tests for a tablet computer, particularly focused on FTD. It consists of twelve different tasks which aim to tap into information processing speed, various aspects of executive function, social cognition, semantic knowledge, calculation and visuospatial skills.

Discussion Future studies will focus on validating the battery in a healthy control cohort, comparing it against a standard 'pen and paper' psychometric battery, and finally testing it within an FTD cohort, including those with genetic forms of FTD where we will be able to assess its ability to detect very early cognitive deficits prior to the onset of symptoms.

Keywords: frontotemporal dementia, progranulin, tau, C9orf72, cognition

For peer review only

STRENGTHS AND LIMITATIONS

- This is the first computerized cognitive battery to focus specifically on FTD.
- Tests are short and can be performed at home, reducing participant burden within studies.
- The study will require validation including comparison with a gold standard ‘pen and paper’ psychometric battery before it can be used more widely.

For peer review only

INTRODUCTION

Frontotemporal dementia (FTD) is a clinically, pathologically and genetically diverse neurodegenerative disease (Warren, Rohrer, & Rossor, 2013). It is probably the commonest form of dementia in those under 60 years of age and is associated with two main clinical presentations: an impairment in personality and social conduct, known as behavioural variant FTD (bvFTD), and deficits in speech and language, called primary progressive aphasia (PPA). Around a third of FTD is familial (Rohrer et al., 2009), with the majority of autosomal dominant FTD linked to mutations in three key genes: chromosome 9 open reading frame 72 (*C9orf72*), progranulin (*GRN*) and microtubule-associated protein tau (*MAPT*).

The cognitive deficits found in FTD are commonly in the executive function and social cognition domains for bvFTD (Grossman, 2002; Harciarek & Jodizo 2005; Harciarek & Cosentino, 2013), and the language and semantic knowledge domains for those with PPA (Harciarek & Cosentino, 2013; Warren, 2013; Woollacott & Rohrer, 2016; Deleon & Miller, 2017), although there is often overlap between these disorders. Classically, episodic memory and more posterior cortical functions are spared, particularly in the early stages of the disease (Gorno-Tempini et al., 2011). The trajectory of cognitive impairment has now been studied across a number of cohorts, with the earliest change being studied mostly within genetic cohorts where at-risk individuals who are many years from expected symptom onset can be investigated. Deficits across several cognitive tasks have been shown around five to eight years prior to symptom onset particularly in tests of executive function and social cognition, although the pattern of deficits can differ according to which mutation is carried (Geschwind et al., 2001, Harciarek & Jodzio, 2005; Hallam et al., 2014; Rohrer et al., 2015; Jiskoot, Dopper, Den Heijer, et al., 2016, Papma et al., 2017; Suhonen et al., 2017, Jiskoot et al., 2018). However, prior studies have been performed mainly using traditional 'pen and paper' tasks which may well be limited in their ability to detect subtle changes. Using magnetic resonance imaging, neuroanatomical changes can be seen in presymptomatic genetic FTD studies much earlier, potentially over

For peer review only - http://bmjopen.bmj.com/site/about/guidelines.xhtml

20 years prior to expected symptom onset (Rohrer et al., 2015), and so there is a need to bridge the gap between the sensitivity of neuroimaging techniques and current cognitive assessments.

Treatment trials in FTD are currently underway, and although many of the planned studies are focused on symptomatic FTD, it is likely that future studies will also include people in the presymptomatic phase of the disease. One of the greatest challenges facing trials of this nature is having robust cognitive outcome measures. The Food and Drug Administration have recognized that current assessments are not optimal, highlighting the need for improved cognitive markers as outcome measures for clinical trials (Sabbagh, Hendrix, & Harrison, 2019), and have encouraged the development of novel approaches to evaluate subtle deficits that may emerge in the presymptomatic stages of dementia (FDA, 2018). Here we describe the design and protocol of Ignite, a novel computerized cognitive assessment tool designed for FTD. We outline the key objectives for developing a computerized battery and provide an overview of the cognitive domains tested within the battery.

METHODS

Patient and Public Involvement

It is standard for traditional cognitive assessments to be performed by trained psychologists in hospital or research settings, which can be both time consuming and costly. On discussion with research participants they describe frequently experiencing high levels of anxiety in relation to attending research visits, particularly in relation to neuropsychological asssessment. In presymptomatic FTD studies this can be most prominent in those who have yet to find out their carrier status. In addition, the number of in person contact days required for a clinical trial can be burdensome and can prevent an individual's participation. With these considerations in mind, we wanted to design a computerized battery that could be performed by

For peer review only

individuals themselves, with the aim of reducing participant anxiety, thus ensuring that their best performance is captured. We therefore initially discussed the development of this battery with a small group of people at-risk of familial FTD to ensure we could maximize the user-friendliness of the battery and reduce the participant burden.

Objectives for developing a computerized cognitive battery

Apart from improving the participant experience, we had a number of other objectives for developing the battery:

Comprehensiveness

We wanted to develop a set of assessments that could capture an individual's performance across a number of cognitive domains in a comprehensive manner for FTD. A major drawback of current cognitive assessments is the duration of testing required to get an accurate picture of individual performance, often taking a number of hours. The new battery was designed to be completed in around 30 minutes or less.

Increased sensitivity

Ignite consists mainly of tests which are modifications of existing cognitive tasks. However, each task was modified to shorten the time allowed for completion, with the measurement of reaction times aiming to increase the sensitivity to detect deficits.

Self-assessment

A key aim in the development of Ignite was its ability to be performed by individuals on their own, ideally while at home. All assessments were designed to be as intuitive as possible, appearing more like short games than standard cognitive tests, including the use of simple on-screen buttons or swipes to record

For peer review only

answers. A challenge in designing tasks with self-assessment in mind is ensuring that participants are motivated to complete the tasks on their own and that they understand what is being asked of them. To assist participants in understanding and remembering task instructions, they are provided with brief written instructions prior to beginning each individual assessment task. Accompanying the written instructions are short videos to ensure participants can clearly visualize and understand what is required of them. In addition, once a task has begun, a prompt of the task instruction is displayed at the top of the screen for the duration of the task. In this way, participants can refresh their memories of the task instructions at any time without having to exit the task. To keep participants motivated and engaged each task was designed to range from 30-180 seconds in duration, with most tasks programmed to time out after 60 seconds.

PROTOCOL

Ignite consists of twelve separate assessments that measure a spectrum of cognitive functions (Table 1, Figure 1). Each test was included based on knowledge of the deficits seen in FTD, and in particular those domains most likely to be affected presymptomatically. The tests are self-administered in a predetermined order, in an environment familiar to the participant, such as their home.

Executive function

The term executive function describes a number of different aspects of cognition including reasoning, problem solving and planning (Collins & Koechlin, 2012) commonly related to the workings of the frontal lobe. It consists of a number of different subdomains, which include inhibitory control, cognitive flexibility and working memory (Diamond, 2013). We aimed to design tasks tapping into each of these subdomains as well as decision making and cognitive timing as previous studies of FTD have shown deficits within all

For peer review only

of them (Strenziok et al., 2011; Manes et al., 2011; Henley et al., 2014), and in particular impairment can be seen presymptomatically across each of the major genetic groups (Rohrer et al., 2015; Staffaroni et al., 2019).

Inhibitory control

This subdomain is described as the ability to control one's attention, behaviour and thoughts to override a strong internal predisposition or external lure (Diamond, 2013). It is abnormal in FTD and relates to the key symptom of disinhibition that forms part of the diagnostic criteria for behavioural variant FTD (Rascovsky et al., 2011). We included two tasks of inhibitory control.

Colour Mix

In our task, we adapted the classical Stroop task including five different colours: red, blue, green, purple and yellow. The initial two levels of the task test speed of processing. In Level 1, participants are presented with a coloured circle and all five colour names written below. They are asked to match the colour of the circle with the correct colour name from 5. They have 30 seconds to complete as many as possible (with a maximum of 50 possible trials). In Level 2, participants are presented with the name of a colour and (as with Level 1) the five colour names written below. They are asked to match the target colour word with the matching word below. They have 30 seconds to complete as many as possible (with a maximum of 50 possible trials). The next two levels measure inhibitory control. In Level 3, participants are presented with a colour word written in a different colour e.g. BLUE written in red ink. They are asked to match the colour of the ink (rather than the name) to one of the five colour names written below. They have 60 seconds to complete as many as possible (with a maximum of 50 possible trials). Lastly, in Level 4, participants are presented as in Level 3 with a colour word written in a different colour, and have to complete the task as in Level 3, unless a black border appears around the word (as it does on some trials), when they have to match the written word rather than the ink colour (e.g. match blue if the word is BLUE written in red ink)

For peer review only - http://bmjopen.bmj.com/site/about/guidelines.xhtml

to one of the five colour names written below. They have 60 seconds to complete as many as possible (with a maximum of 50 possible trials).

Swipe Out

Our task was a modification of the classical Flanker task, with an arrowhead (facing up, down, left or right) with four flanking arrowheads surrounding the target arrowhead. Participants are asked to swipe in the direction of the central arrowhead. In some trials the flanking arrowheads are facing in the same direction, and in some trials they are facing in the opposite direction. Participants have 60 seconds to complete as many trials as possible (with a maximum of 40 possible trials).

Cognitive flexibility

This subdomain describes the ability to change perspectives from an established pattern, and mentally adjust or 'set shift' our perspective to new task demands. Tasks that tap into cognitive flexibility include the Wisconsin Card Sorting Task (WCST) (Stuss et al., 2000) and the Trail Making Test (TMT) (Delis et al., 2001). Deficits in both of these tasks have been previously described in FTD (Geschwind et al., 2001; Rohrer et al., 2008; Jiskoot et al., 2016) including presymptomatically in those with genetic forms of FTD (Barandiaran, et al., 2012).

Card Sort

Our task adapts a classical card sorting task, asking participants to sort cards by dragging them from the centre of the screen into card deposits located in the four corners of the screen. Participants are required to sort cards by a rule: either shape, number or colour. The primary aim of the task is for the participant to understand the correct sorting criteria on the basis of feedback, either correct or incorrect, and to switch flexibly between sorting rules whenever feedback indicates that the rule has changed. After a set number

For peer review only

of correct card sorts are performed the rule changes, and negative feedback is given if participants continue to sort by the previous rule. The successful performance of the task is measured in participants' ability to flexibly respond to feedback given in shifting to the new rule (Stuss et al., 2000). The number of perseverative errors, identified by participants failing to adapt to a new rule, is thought to be indicative of poor cognitive flexibility (Lange, Brückner, Knebel, Seer, & Kopp, 2018). In our task, participants have a total of 90 seconds in order to sort as many as cards as possible.

Path Finder

This task adapts the Trail Making Test (TMT), asking participants to tap stimuli in sequential order (rather than draw an unbroken line between letters and/or numbers as in the original pen and paper task). Correct sequencing is indicated by the outline of each letter or number changing colour to green. The letter or number changes to red if selected incorrectly. In Level 1 participants are asked to select numbers in the correct order (similar to TMT part A), with a maximum of 60 seconds to complete the task. In Level 2 participants are asked to alternate between numbers and letters in the correct order (similar to TMT part B), with a maximum of 90 seconds to complete the task.

Working memory

This subdomain of executive function describes the ability to hold information in mind and mentally work with it (Baddeley & Hitch, 1994). A classic paradigm for assessing working memory is the N-back task, which requires participants to monitor a series of stimuli and respond whenever the stimuli presented matches the properties of the trial presented 'N' before it (Owen, McMillan, Laird, & Bullmore, 2005). The task requires the monitoring, updating and manipulation of information displayed to perform the task effectively.

For peer review only

Think Back

There are two levels to our N-Back task. Level 1 is a 1-back task and Level 2 is a 2-back task. In Level 1 participants must indicate whether a shape matches the one preceding it. In Level 2 participants must indicate whether a shape matches the shape shown two before it, intended to place greater demand on working memory. An answer is submitted by pressing a 'yes' or 'no' button. In each level, participants have 60 seconds to complete as many trials as possible (up to a maximum of 72 trials).

Decision making

For many people with FTD their ability to make decisions is compromised (Gleichgerrcht et al., 2012). Several paradigms are employed in assessing decision making behaviour, often in the form of gambling or risk-taking tasks, such as the Iowa Gambling Task (Bechara & Van der Linden, 2005), Cambridge Gambling Task (Rogers et al., 1999), Game of Dice Task (Brand et al., 2005) and the Balloon Analogue Risk Task (Lejuez et al., 2002), with people with FTD shown to be impaired on these tests (Rahman, Sahakian, Hodges, Rogers, & Robbins, 1999).

Balloon Fair

In our task we adapted the Balloon Analogue Risk Task (Lejuez et al., 2002) to assess decision making behaviour. Participants are presented with a simulated balloon and balloon pump. Also presented is a button labelled 'Collect £' and a permanent money counter labelled 'Total Earned', alongside a counter displaying money that has been lost. With each press on the pump, the balloon is inflated, and money is accumulated in a temporary bank. Balloons pop after a predetermined 'popping' point, at which point the money accrued in the temporary bank is lost. Participants have the option to cash in the money accumulated in the temporary bank at any point by clicking the 'Collect £' button. After each balloon has popped (money lost) or the money has been collected a new balloon appears. Balloons are differently

For peer review only

coloured (green, yellow and blue) with each colour associated with a unique sum (£5, £10, £50 respectively), and a unique popping point (quicker for higher sums). Participants are unaware of the probability of a balloon popping. The aim of the task is to earn as much money as possible. Participants have 90 seconds to complete the task.

Cognitive timing

The concept of timing is integral to performing many mental processes (Allman & Meck, 2012). Although not well-studied in FTD, one previous study has shown impairment in both externally-paced and self-paced finger tapping (Henley et al., 2014).

Time Tap

We adapted the task described in Henley et al., 2014 to test cognitive timing. A pulsating circle is presented in the middle of the top of the screen and a tone sounds in time to the pulse (at 1500ms intervals). Participants are asked to use the index finger of their dominant hand to tap in time with the pulsating circle on a second circle presented below. After 30 seconds the pulsation and tone cease, and participants are instructed to maintain the same tempo for a further 30 seconds.

Social Cognition

Social cognition is the ability to perceive, interpret and generate a response to the intentions, behaviours, and feelings of others (Frith, 2008), and includes a number of subdomains such as emotion recognition (Ekman et al., 1987) and theory of mind (Premack & Woodruff, 1978; Baron-Cohen et al., 1997). Social cognition impairment occurs early and almost universally in all people with FTD (Snowden et al., 2003; Harciarek and Cosentino, 2013; Russell et al., 2020) as well as those with other forms of FTD, such as PPA (Couto et al., 2013; Fittipaldi et al., 2019). These deficits include poor recognition of simple emotions in faces

For peer review only

(Kumfor & Piguet, 2012; Lough et al., 2006), as well as impairment of complex emotion processing, and other tasks of theory of mind (Gregory et al., 2002; Snowden et al., 2003; Lough et al., 2006). Early deficits have been seen in genetic FTD including those who are presymptomatic (Jiskoot, Dopper, Heijer, et al., 2016).

Face Match

In our task of simple emotion processing participants are presented with a target basic emotion word (sadness, happiness, anger, fear, disgust, surprise) at the top of the screen alongside nine images of faces in the corners of the screen. Participants are asked to tap all of the faces displaying the target emotion as quickly as possible. There are five correct answers out of each nine presented faces, and participants must press all five within 10 seconds, or the task moves on to the next set of nine faces. The participants have to complete as many items as possible within 60 seconds (up to a maximum of 6 sets of nine faces).

Mind Reading

Our task of complex emotion processing adapts the Reading the Mind in the Eyes test so that participants are presented with a target emotion word in the centre of the screen alongside four images of eyes in the corners of the screen, one of which matches the emotion. Participants are asked to select the image which best matches the target emotion word, completing as many as possible within 90 seconds (up to a maximum of 20 words).

Semantic knowledge

Impairment of semantic knowledge is characteristic of the semantic variant of primary progressive aphasia (Gorno-Tempini et al., 2011*)* but is seen in other forms of FTD, and has been shown to occur

For peer review only

presymptomatically, particularly in those with *MAPT* mutations (Snowden et al., 2015; Rohrer et al., 2015; Hardy et al., 2016, Jiskoot et al., 2016).

Picture Pair

Our task uses stimuli from the modified version of the Camel and Cactus task that we have previously described as detecting early semantic deficits in genetic FTD (Moore et al., 2020). The participant has to match a target picture with a semantically-linked matching picture from a choice of four e.g. matching Camel with Cactus rather than a tree, sunflower or rose. The participants have to answer as many questions as possible in 120 seconds (with a maximum of 25 questions).

Arithmetic

Calculation abilities have been shown to be highly dependent on left parietal lobe function (Takayama et al., 1994; Dehaene, Piazza, Pinel, & Cohen, 2003). Although initial studies focused on anterior involvement of the frontal and temporal lobes in FTD, more recent studies have shown that some forms of FTD can have more posterior cortical atrophy, particularly those with *GRN* mutations and *C9orf72* expansions (Cash et al., 2018), including presymptomatically (Rohrer et al., 2015). Dyscalculia has been reported in both of these groups (Rohrer, Warren, et al., 2008; Mahoney, Beck, et al., 2012). Although deficits are generally not limited to one form of mental arithmetic, one study has shown deficits specifically in multiplication in ALS (Palmieri et al., 2013).

Sum Up

Our task includes additions, subtractions, multiplications and divisions, with participants presented with the sum and four possible answers, and a time limit of 10 seconds for completion of each sum. The

For peer review only

participants have to answer as many questions as possible within 60 seconds (with a maximum of 48 sums, 12 of each type).

Visuospatial skills

Visuospatial processing is classically associated with right parietal lobe function (Rohrer et al., 2008; Bartolomeo et al., 2012). As mentioned above, more posterior cortical atrophy, including involvement of the right parietal lobe has been seen in genetic FTD. Given this finding, it is unsurprising that several studies have highlighted impairments of visuospatial skills in both *GRN* mutation carriers (Taipa et al., 2012; Hallam et al., 2014; Taghdiri et al., 2016) and *C9orf72* expansion carriers (Patel & Sampson, 2015).

Line Judge

Judgement of line orientation is a standard measure of visuospatial skills (Benton, Varney & Hamsher, 1983; Qualls, Bliwise, & Stringer, 2000), testing an individual's ability to match the angle and orientation of lines in space, with deficits seen in forms of dementia such as Alzheimer's diseases and Dementia with Lewy Bodies which are usually associated with parietal atrophy (Simard et al., 2003; Salimi et al., 2018).

In our task, participants are presented with pairs of angled lines and are asked to match the target pair of lines to a set of 11 lines arranged in a semicircle and numbered 1 to 11. Participants press the correct numbers that correspond to the line pair. The participants have to answer as many questions as possible within a 90 second timeframe (up to a maximum of 15 pairs).

Discussion

Ignite is a novel computerized battery of assessments for the detection of cognitive changes in FTD. It includes tasks of information processing speed, executive function, social cognition, semantic knowledge,

For peer review only

arithmetic and visuospatial skills. It potentially may be sensitive enough to detect early (and presymptomatic) deficits within an at-risk genetic cohort. Ignite will be initially validated in a healthy control cohort and compared against a standard set of 'pen and paper' psychometric tasks, including assessment of test-retest reliability. The battery will then be tested within an FTD cohort such as GENFI (www.genfi.org; Rohrer et al., 2015) where it would be possible to assess both presymptomatic and symptomatic people with FTD.

Conclusions

The final goal of the Ignite study will be to develop a set of validated tasks which are able to track disease progression in FTD, and therefore have the potential to be employed in clinical trials as a cognitive outcome measure.

Abbreviations

FTD, frontotemporal dementia; PPA, primary progressive aphasia; C9orf72, chromosome 9 open reading frame 72; GRN, progranulin; MAPT, microtubule-associated protein tau; bvFTD, behavioural variant frontotemporal dementia, TMT, Trail Making Test; WCST, Wisconsin Card Sorting Task.

For peer review only

Declarations

Ethics approval and consent to participate

The Ignite study has been approved by the UCL ethics committee. Written informed consent is being obtained for those taking part in the study.

Consent to publish

Not applicable

Availability of data and materials

The Ignite app is free to download and use from the Appstore.

Competing interests

KMM is now an employee of Ionis Pharmaceuticals. JDR has served on medical advisory boards or provided consultancy for Alector, Arkuda Therapeutics, Wave Life Sciences, Prevail Therapeutics, UCB, AC Immune, Astex Pharmaceuticals, Biogen, Takeda and Eisai.

Funding

The app was developed initially through an Alzheimer's Society PhD studentship (for KMM) awarded to JDR (AS-PhD-2015-005). RSC has been funded by a Frontotemporal Dementia Research Studentship in Memory of David Blechner awarded through The National Brain Appeal (RCN 290173). JDR has received funding from an MRC Clinician Scientist Fellowship (MR/M008525/1), an NIHR Rare Disease Translational Research Collaboration (BRC149/NS/MH) and the Brain Research UK Miriam Marks Senior Fellowship; his work is also supported by the MRC UK GENFI grant (MR/M023664/1), the Bluefield Project and the JPND GENFI-PROX grant (2019-02248). None of the funding bodies had any direct input into the app development.

Authors' contributions

KMM, RSC and JDR wrote the manuscript. All authors have read and approved the manuscript.

Acknowledgements

We thank the research participants at the Dementia Research Centre for their contribution to the development of Ignite.

For peer review only

References

1. Warren, J. D., Rohrer, J. D., & Rossor, M. N. (2013). Clinical review. Frontotemporal dementia. BMJ (Clinical research ed.), 347, f4827. https://doi.org/10.1136/bmj.f4827
2. Rohrer, J. D., Guerreiro, R., Vandrovcova, J., Uphill, J., Reiman, D., Beck, J., Isaacs, A. M., Authier, A., Ferrari, R., Fox, N. C., Mackenzie, I. R., Warren, J. D., de Silva, R., Holton, J., Revesz, T., Hardy, J., Mead, S., & Rossor, M. N. (2009). The heritability and genetics of frontotemporal lobar degeneration. Neurology, 73(18), 1451–1456. https://doi.org/10.1212/WNL.0b013e3181bf997a
3. Grossman M. (2002). Frontotemporal dementia: a review. Journal of the International Neuropsychological Society : JINS, 8(4), 566–583. https://doi.org/10.1017/s1355617702814357
4. Harciarek, M., & Jodzio, K. (2005). Neuropsychological differences between frontotemporal dementia and Alzheimer's disease: a review. Neuropsychology review, 15(3), 131–145. https://doi.org/10.1007/s11065-005-7093-4
5. Harciarek, M., & Cosentino, S. (2013). Language, executive function and social cognition in the diagnosis of frontotemporal dementia syndromes. International review of psychiatry (Abingdon, England), 25(2), 178–196. https://doi.org/10.3109/09540261.2013.763340
6. Warren, J. D., Rohrer, J. D., & Rossor, M. N. (2013). Clinical review. Frontotemporal dementia. BMJ (Clinical research ed.), 347, f4827. https://doi.org/10.1136/bmj.f4827
7. Woollacott, I. O., & Rohrer, J. D. (2016). The clinical spectrum of sporadic and familial forms of frontotemporal dementia. Journal of neurochemistry, 138 Suppl 1, 6–31. https://doi.org/10.1111/jnc.13654
8. Deleon, J., & Miller, B. L. (2018). Frontotemporal dementia. Handbook of clinical neurology, 148, 409–430. https://doi.org/10.1016/B978-0-444-64076-5.00027-2.
9. Gorno-Tempini, M. L., Hillis, A. E., Weintraub, S., Kertesz, A., Mendez, M., Cappa, S. F., Ogar, J. M., Rohrer, J. D., Black, S., Boeve, B. F., Manes, F., Dronkers, N. F., Vandenberghe, R., Rascovsky,

For peer review only

K., Patterson, K., Miller, B. L., Knopman, D. S., Hodges, J. R., Mesulam, M. M., & Grossman, M. (2011). Classification of primary progressive aphasia and its variants. Neurology, 76(11), 1006–1014. https://doi.org/10.1212/WNL.0b013e31821103e6

10. Geschwind, D. H., Robidoux, J., Alarcón, M., Miller, B. L., Wilhelmsen, K. C., Cummings, J. L., & Nasreddine, Z. S. (2001). Dementia and neurodevelopmental predisposition: cognitive dysfunction in presymptomatic subjects precedes dementia by decades in frontotemporal dementia. Annals of neurology, 50(6), 741–746. https://doi.org/10.1002/ana.10024

11. Hallam, B. J., Jacova, C., Hsiung, G. Y., Wittenberg, D., Sengdy, P., Bouchard-Kerr, P., Slack, P., Rademakers, R., Baker, M., Chow, T. W., Levine, B., Feldman, H. H., & Mackenzie, I. R. (2014). Early neuropsychological characteristics of progranulin mutation carriers. Journal of the International Neuropsychological Society : JINS, 20(7), 694–703. https://doi.org/10.1017/S1355617714000551

12. Rohrer, J. D., Nicholas, J. M., Cash, D. M., van Swieten, J., Dopper, E., Jiskoot, L., van Minkelen, R., Rombouts, S. A., Cardoso, M. J., Clegg, S., Espak, M., Mead, S., Thomas, D. L., De Vita, E., Masellis, M., Black, S. E., Freedman, M., Keren, R., MacIntosh, B. J., Rogaeva, E., … Binetti, G. (2015). Presymptomatic cognitive and neuroanatomical changes in genetic frontotemporal dementia in the Genetic Frontotemporal dementia Initiative (GENFI) study: a cross-sectional analysis. The Lancet. Neurology, 14(3), 253–262. https://doi.org/10.1016/S1474-4422(14)70324-2

13. Jiskoot, L. C., Dopper, E. G., Heijer, T. d., Timman, R., van Minkelen, R., van Swieten, J. C., & Papma, J. M. (2016). Presymptomatic cognitive decline in familial frontotemporal dementia: A longitudinal study. Neurology, 87(4), 384–391. https://doi.org/10.1212/WNL.0000000000002895

14. Papma, J. M., Jiskoot, L. C., Panman, J. L., Dopper, E. G., den Heijer, T., Donker Kaat, L., Pijnenburg, Y., Meeter, L. H., van Minkelen, R., Rombouts, S., & van Swieten, J. C. (2017). Cognition and gray

For peer review only

and white matter characteristics of presymptomatic C9orf72 repeat expansion. Neurology, 89(12), 1256–1264. https://doi.org/10.1212/WNL.0000000000004393

15. Suhonen, N. M., Haanpää, R. M., Korhonen, V., Jokelainen, J., Pitkäniemi, A., Heikkinen, A. L., Krüger, J., Hartikainen, P., Helisalmi, S., Hiltunen, M., Hänninen, T., & Remes, A. M. (2017). Neuropsychological Profile in the C9ORF72 Associated Behavioral Variant Frontotemporal Dementia. Journal of Alzheimer's disease : JAD, 58(2), 479–489. https://doi.org/10.3233/JAD-161142

16. Jiskoot, L. C., Panman, J. L., van Asseldonk, L., Franzen, S., Meeter, L., Donker Kaat, L., van der Ende, E. L., Dopper, E., Timman, R., van Minkelen, R., van Swieten, J. C., van den Berg, E., & Papma, J. M. (2018). Longitudinal cognitive biomarkers predicting symptom onset in presymptomatic frontotemporal dementia. Journal of neurology, 265(6), 1381–1392. https://doi.org/10.1007/s00415-018-8850-7

17. Sabbagh, M. N., Hendrix, S., & Harrison, J. E. (2019). FDA position statement "Early Alzheimer's disease: Developing drugs for treatment, Guidance for Industry". Alzheimer's & dementia (New York, N. Y.), 5, 13–19. https://doi.org/10.1016/j.trci.2018.11.004

18. FDA (2018). Early Alzheimer’s Disease: Developing Drugs for Treatment Guidance for Industry DRAFT GUIDANCE. Retrieved from https://www.fda.gov/Drugs/GuidanceComplianceRegulatoryInformation/Guidances/default.htm

19. Collins, A., & Koechlin, E. (2012). Reasoning, learning, and creativity: frontal lobe function and human decision-making. PLoS biology, 10(3), e1001293. https://doi.org/10.1371/journal.pbio.1001293

20. Diamond A. (2013). Executive functions. Annual review of psychology, 64, 135–168. https://doi.org/10.1146/annurev-psych-113011-143750

21. Strenziok, M., Pulaski, S., Krueger, F., Zamboni, G., Clawson, D., & Grafman, J. (2011). Regional brain atrophy and impaired decision making on the balloon analog risk task in behavioral variant

For peer review only

frontotemporal dementia. Cognitive and behavioral neurology : official journal of the Society for Behavioral and Cognitive Neurology, 24(2), 59–67. https://doi.org/10.1097/WNN.0b013e3182255a7c

22. Manes, F., Torralva, T., Ibáñez, A., Roca, M., Bekinschtein, T., & Gleichgerrcht, E. (2011). Decision-making in frontotemporal dementia: clinical, theoretical and legal implications. Dementia and geriatric cognitive disorders, 32(1), 11–17. https://doi.org/10.1159/000329912

23. Henley, S. M., Downey, L. E., Nicholas, J. M., Kinnunen, K. M., Golden, H. L., Buckley, A., Mahoney, C. J., & Crutch, S. J. (2014). Degradation of cognitive timing mechanisms in behavioural variant frontotemporal dementia. Neuropsychologia, 65, 88–101. https://doi.org/10.1016/j.neuropsychologia.2014.10.009

24. Staffaroni, A. M., Bajorek, L., Casaletto, K. B., Cobigo, Y., Goh, S. M., Wolf, A., Heuer, H. W., Elahi, F. M., Ljubenkov, P. A., Dever, R., Kornak, J., Appleby, B., Bove, J., Bordelon, Y., Brannelly, P., Brushaber, D., Caso, C., Coppola, G., Dheel, C., Dickerson, B. C., … ARTFL/LEFFTDS consortium (2020). Assessment of executive function declines in presymptomatic and mildly symptomatic familial frontotemporal dementia: NIH-EXAMINER as a potential clinical trial endpoint. Alzheimer's & dementia : the journal of the Alzheimer's Association, 16(1), 11–21. https://doi.org/10.1016/j.jalz.2019.01.012

25. Rascovsky, K., Hodges, J. R., Knopman, D., Mendez, M. F., Kramer, J. H., Neuhaus, J., van Swieten, J. C., Seelaar, H., Dopper, E. G., Onyike, C. U., Hillis, A. E., Josephs, K. A., Boeve, B. F., Kertesz, A., Seeley, W. W., Rankin, K. P., Johnson, J. K., Gorno-Tempini, M. L., Rosen, H., Prioleau-Latham, C. E., … Miller, B. L. (2011). Sensitivity of revised diagnostic criteria for the behavioural variant of frontotemporal dementia. Brain : a journal of neurology, 134(Pt 9), 2456–2477. https://doi.org/10.1093/brain/awr179

For peer review only

26. Stuss, D. T., Levine, B., Alexander, M. P., Hong, J., Palumbo, C., Hamer, L., Murphy, K. J., & Izukawa, D. (2000). Wisconsin Card Sorting Test performance in patients with focal frontal and posterior brain damage: effects of lesion location and test structure on separable cognitive processes. Neuropsychologia, 38(4), 388–402. https://doi.org/10.1016/s0028-3932(99)00093-7

27. Delis, D. ., Kaplan, E., & J.H, K. (2001). The Delis-Kaplan Executive Function System: Examiner's Manual. San Andonio: The Psychological Corporation.

28. Rohrer, J. D., Warren, J. D., Omar, R., Mead, S., Beck, J., Revesz, T., Holton, J., Stevens, J. M., Al-Sarraj, S., Pickering-Brown, S. M., Hardy, J., Fox, N. C., Collinge, J., Warrington, E. K., & Rossor, M. N. (2008). Parietal lobe deficits in frontotemporal lobar degeneration caused by a mutation in the progranulin gene. Archives of neurology, 65(4), 506–513. https://doi.org/10.1001/archneur.65.4.506

29. Barandiaran, M., Estanga, A., Moreno, F., Indakoetxea, B., Alzualde, A., Balluerka, N., Martí Massó, J. F., & López de Munain, A. (2012). Neuropsychological features of asymptomatic c.709-1G>A progranulin mutation carriers. Journal of the International Neuropsychological Society : JINS, 18(6), 1086–1090. https://doi.org/10.1017/S1355617712000823

30. Lange, F., Brückner, C., Knebel, A., Seer, C., & Kopp, B. (2018). Executive dysfunction in Parkinson's disease: A meta-analysis on the Wisconsin Card Sorting Test literature. Neuroscience and biobehavioral reviews, 93, 38–56. https://doi.org/10.1016/j.neubiorev.2018.06.014

31. Baddeley A.D, & Hitch G.J. (1994). Developments in the Concept of Working Memory. Neuropsychology, 8, 485–493. Retrieved from http://ovidsp.dc2.ovid.com.libproxy.ucl.ac.uk/sp-4.01.0a/ovidweb.cgi?WebLinkFrameset=1&S=HDKHFPIJFOEBDLPOJPCKFGBHMKCEAA00&returnUrl=ovidweb.cgi%3F%26Full%2BText%3DL%257cS.sh.22.23%257c0%257c00062896-199410000-00002%26S%3DHDKHFPIJFOEBDLPOJPCKFGBHMKCEAA00&d

For peer review only

32. Owen, A. M., McMillan, K. M., Laird, A. R., & Bullmore, E. (2005). N-back working memory paradigm: a meta-analysis of normative functional neuroimaging studies. Human brain mapping, 25(1), 46–59. https://doi.org/10.1002/hbm.20131

33. Gleichgerrcht, E., Torralva, T., Roca, M., Szenkman, D., Ibanez, A., Richly, P., Pose, M., & Manes, F. (2012). Decision making cognition in primary progressive aphasia. Behavioural neurology, 25(1), 45–52. https://doi.org/10.3233/BEN-2012-0352

34. Bechara, A., & Van Der Linden, M. (2005). Decision-making and impulse control after frontal lobe injuries. Current opinion in neurology, 18(6), 734–739. https://doi.org/10.1097/01.wco.0000194141.56429.3c

35. Rogers, R. D., Owen, A. M., Middleton, H. C., Williams, E. J., Pickard, J. D., Sahakian, B. J., & Robbins, T. W. (1999). Choosing between small, likely rewards and large, unlikely rewards activates inferior and orbital prefrontal cortex. The Journal of neuroscience : the official journal of the Society for Neuroscience, 19(20), 9029–9038. https://doi.org/10.1523/JNEUROSCI.19-20-09029.1999

36. Brand, M., Fujiwara, E., Borsutzky, S., Kalbe, E., Kessler, J., & Markowitsch, H. J. (2005). Decision-making deficits of korsakoff patients in a new gambling task with explicit rules: associations with executive functions. Neuropsychology, 19(3), 267–277. https://doi.org/10.1037/0894-4105.19.3.267

37. Lejuez, C. W., Read, J. P., Kahler, C. W., Richards, J. B., Ramsey, S. E., Stuart, G. L., Strong, D. R., & Brown, R. A. (2002). Evaluation of a behavioral measure of risk taking: the Balloon Analogue Risk Task (BART). Journal of experimental psychology. Applied, 8(2), 75–84. https://doi.org/10.1037//1076-898x.8.2.75

38. Rahman, S., Sahakian, B. J., Hodges, J. R., Rogers, R. D., & Robbins, T. W. (1999). Specific cognitive deficits in mild frontal variant frontotemporal dementia. Brain : a journal of neurology, 122 (Pt 8), 1469–1493. https://doi.org/10.1093/brain/122.8.1469

For peer review only

39. Allman, M. J., & Meck, W. H. (2012). Pathophysiological distortions in time perception and timed performance. Brain : a journal of neurology, 135(Pt 3), 656–677. https://doi.org/10.1093/brain/awr210

40. Snowden, J. S., Gibbons, Z. C., Blackshaw, A., Doubleday, E., Thompson, J., Craufurd, D., Foster, J., Happé, F., & Neary, D. (2003). Social cognition in frontotemporal dementia and Huntington's disease. Neuropsychologia, 41(6), 688–701. https://doi.org/10.1016/s0028-3932(02)00221-x

41. Russell, L. L., Greaves, C. V., Bocchetta, M., Nicholas, J., Convery, R. S., Moore, K., Cash, D. M., van Swieten, J., Jiskoot, L., Moreno, F., Sanchez-Valle, R., Borroni, B., Laforce, R., Jr, Masellis, M., Tartaglia, M. C., Graff, C., Rotondo, E., Galimberti, D., Rowe, J. B., Finger, E., … Genetic FTD Initiative, GENFI (2020). Social cognition impairment in genetic frontotemporal dementia within the GENFI cohort. Cortex; a journal devoted to the study of the nervous system and behavior, 133, 384–398. https://doi.org/10.1016/j.cortex.2020.08.023

42. Couto, B., Manes, F., Montañés, P., Matallana, D., Reyes, P., Velasquez, M., Yoris, A., Baez, S., & Ibáñez, A. (2013). Structural neuroimaging of social cognition in progressive non-fluent aphasia and behavioral variant of frontotemporal dementia. Frontiers in human neuroscience, 7, 467. https://doi.org/10.3389/fnhum.2013.00467

43. Fittipaldi, S., Ibanez, A., Baez, S., Manes, F., Sedeno, L., & Garcia, A. M. (2019). More than words: Social cognition across variants of primary progressive aphasia. Neuroscience and biobehavioral reviews, 100, 263–284. https://doi.org/10.1016/j.neubiorev.2019.02.020

44. Kumfor, F., & Piguet, O. (2012). Disturbance of emotion processing in frontotemporal dementia: a synthesis of cognitive and neuroimaging findings. Neuropsychology review, 22(3), 280–297. https://doi.org/10.1007/s11065-012-9201-6

For peer review only

45. Lough, S., Kipps, C. M., Treise, C., Watson, P., Blair, J. R., & Hodges, J. R. (2006). Social reasoning, emotion and empathy in frontotemporal dementia. Neuropsychologia, 44(6), 950–958. https://doi.org/10.1016/j.neuropsychologia.2005.08.009

46. Gregory, C., Lough, S., Stone, V., Erzinclioglu, S., Martin, L., Baron-Cohen, S., & Hodges, J. R. (2002). Theory of mind in patients with frontal variant frontotemporal dementia and Alzheimer's disease: theoretical and practical implications. Brain : a journal of neurology, 125(Pt 4), 752–764. https://doi.org/10.1093/brain/awf079

47. Tottenham, N., Tanaka, J. W., Leon, A. C., McCarry, T., Nurse, M., Hare, T. A., Marcus, D. J., Westerlund, A., Casey, B. J., & Nelson, C. (2009). The NimStim set of facial expressions: judgments from untrained research participants. Psychiatry research, 168(3), 242–249. https://doi.org/10.1016/j.psychres.2008.05.006

48. Snowden, J. S., Adams, J., Harris, J., Thompson, J. C., Rollinson, S., Richardson, A., Jones, M., Neary, D., Mann, D. M., & Pickering-Brown, S. (2015). Distinct clinical and pathological phenotypes in frontotemporal dementia associated with MAPT, PGRN and C9orf72 mutations. Amyotrophic lateral sclerosis & frontotemporal degeneration, 16(7-8), 497–505. https://doi.org/10.3109/21678421.2015.1074700

49. Hardy, C. J., Buckley, A. H., Downey, L. E., Lehmann, M., Zimmerer, V. C., Varley, R. A., Crutch, S. J., Rohrer, J. D., Warrington, E. K., & Warren, J. D. (2016). The Language Profile of Behavioral Variant Frontotemporal Dementia. Journal of Alzheimer's disease : JAD, 50(2), 359–371. https://doi.org/10.3233/JAD-150806

50. Moore, K., Convery, R., Bocchetta, M., Neason, M., Cash, D. M., Greaves, C., Russell, L. L., Clarke, M., Peakman, G., van Swieten, J., Jiskoot, L., Moreno, F., Barandiaran, M., Sanchez-Valle, R., Borroni, B., Laforce, R., Jr, Doré, M. C., Masellis, M., Tartaglia, M. C., Graff, C., … Genetic FTD Initiative, GENFI* (2020). A modified Camel and Cactus Test detects presymptomatic semantic

For peer review only

impairment in genetic frontotemporal dementia within the GENFI cohort. Applied neuropsychology. Adult, 1–8. Advance online publication. https://doi.org/10.1080/23279095.2020.1716357

51. Takayama, Y., Sugishita, M., Akiguchi, I., & Kimura, J. (1994). Isolated acalculia due to left parietal lesion. Archives of neurology, 51(3), 286–291. https://doi.org/10.1001/archneur.1994.00540150084021

52. Dehaene, S., Piazza, M., Pinel, P., & Cohen, L. (2003). Three parietal circuits for number processing. Cognitive neuropsychology, 20(3), 487–506. https://doi.org/10.1080/02643290244000239

53. Cash, D. M., Bocchetta, M., Thomas, D. L., Dick, K. M., van Swieten, J. C., Borroni, B., Galimberti, D., Masellis, M., Tartaglia, M. C., Rowe, J. B., Graff, C., Tagliavini, F., Frisoni, G. B., Laforce, R., Jr, Finger, E., de Mendonça, A., Sorbi, S., Rossor, M. N., Ourselin, S., Rohrer, J. D., … Genetic FTD Initiative, GENFI (2018). Patterns of gray matter atrophy in genetic frontotemporal dementia: results from the GENFI study. Neurobiology of aging, 62, 191–196. https://doi.org/10.1016/j.neurobiolaging.2017.10.008

54. Rohrer, J. D., Warren, J. D., Barnes, J., Mead, S., Beck, J., Pepple, T., Boyes, R., Omar, R., Collinge, J., Stevens, J. M., Warrington, E. K., Rossor, M. N., & Fox, N. C. (2008). Mapping the progression of progranulin-associated frontotemporal lobar degeneration. Nature clinical practice. Neurology, 4(8), 455–460. https://doi.org/10.1038/ncpneuro0869

55. Mahoney, C. J., Downey, L. E., Ridgway, G. R., Beck, J., Clegg, S., Blair, M., Finnegan, S., Leung, K. K., Yeatman, T., Golden, H., Mead, S., Rohrer, J. D., Fox, N. C., & Warren, J. D. (2012). Longitudinal neuroimaging and neuropsychological profiles of frontotemporal dementia with C9ORF72 expansions. Alzheimer's research & therapy, 4(5), 41. https://doi.org/10.1186/alzrt144

56. Palmieri, A., Sorarù, G., D'Ascenzo, C., Balestreri, S., Arcara, G., Ermani, M., Pegoraro, E., & Semenza, C. (2013). Specific numerical processing impairment in ALS patients. Amyotrophic

For peer review only

lateral sclerosis & frontotemporal degeneration, 14(1), 6–12. https://doi.org/10.3109/17482968.2012.719235

57. Bartolomeo, P., Thiebaut de Schotten, M., & Chica, A. B. (2012). Brain networks of visuospatial attention and their disruption in visual neglect. Frontiers in human neuroscience, 6, 110. https://doi.org/10.3389/fnhum.2012.00110

58. Taipa, R., Tuna, A., Damásio, J., Pinto, P. S., Cavaco, S., Pereira, S., Milterberger-Miltenyi, G., Galimberti, D., & Melo-Pires, M. (2012). Clinical, neuropathological, and genetic characteristics of the novel IVS9+1delG GRN mutation in a patient with frontotemporal dementia. Journal of Alzheimer's disease : JAD, 30(1), 83–90. https://doi.org/10.3233/JAD-2012-112084

59. Taghdiri, F., Sato, C., Ghani, M., Moreno, D., Rogaeva, E., & Tartaglia, M. C. (2016). Novel GRN Mutations in Patients with Corticobasal Syndrome. Scientific reports, 6, 22913. https://doi.org/10.1038/srep22913

60. Patel, A. N., & Sampson, J. B. (2015). Cognitive Profile of C9orf72 in Frontotemporal Dementia and Amyotrophic Lateral Sclerosis. Current neurology and neuroscience reports, 15(9), 59. https://doi.org/10.1007/s11910-015-0582-9

61. Benton, A. L., Hamsher, K., Varney, N. R., & Spreen, O. (1983). Contributions to neuropsychological assessment. New York: Oxford University Press.

62. Qualls, C. E., Bliwise, N. G., & Stringer, A. Y. (2000). Short forms of the Benton judgment of line orientation test: Development and psychometric properties. Archives of Clinical Neuropsychology, 15(2), 159–163.

63. Simard, M., van Reekum, R., & Myran, D. (2003). Visuospatial impairment in dementia with Lewy bodies and Alzheimer's disease: a process analysis approach. International journal of geriatric psychiatry, 18(5), 387–391. https://doi.org/10.1002/gps.839

For peer review only

64. Salimi, S., Irish, M., Foxe, D., Hodges, J. R., Piguet, O., & Burrell, J. R. (2017). Can visuospatial measures improve the diagnosis of Alzheimer's disease?. Alzheimer's & dementia (Amsterdam, Netherlands), 10, 66–74. https://doi.org/10.1016/j.dadm.2017.10.004

Figure Legends

Figure 1: Examples of tasks in the Ignite battery: top row left *Colour Mix*, top row middle *Swipe Out*, top row right *Card Sort*, middle row left *Path Finder*, middle row middle *Think Back*, middle row right *Balloon Fair*, bottom row left *Picture Pair*, bottom row middle *Sum Up*, bottom row right *Line Judge*.

Table 1: Summary of tasks within the Ignite protocol

	**Cognitive domain**	**Cognitive subdomain**	**Task**
1	Executive function and information processing speed	Inhibitory control	Colour Mix
**2**			Swipe Out
**3**		Cognitive flexibility	Card Sort
**4**			Path Finder
**5**		Working memory	Think Back
**6**		Decision making	Balloon Fair
**7**		Cognitive Timing	Time Tap
**8**	Social cognition	Emotion processing	Face Match
**9**		Theory of Mind	Mind Reading
**10**	Semantic knowledge		Picture Pair
**11**	Arithmetic		Sum Up
**12**	Visuospatial skills		Line Judge

For peer review only

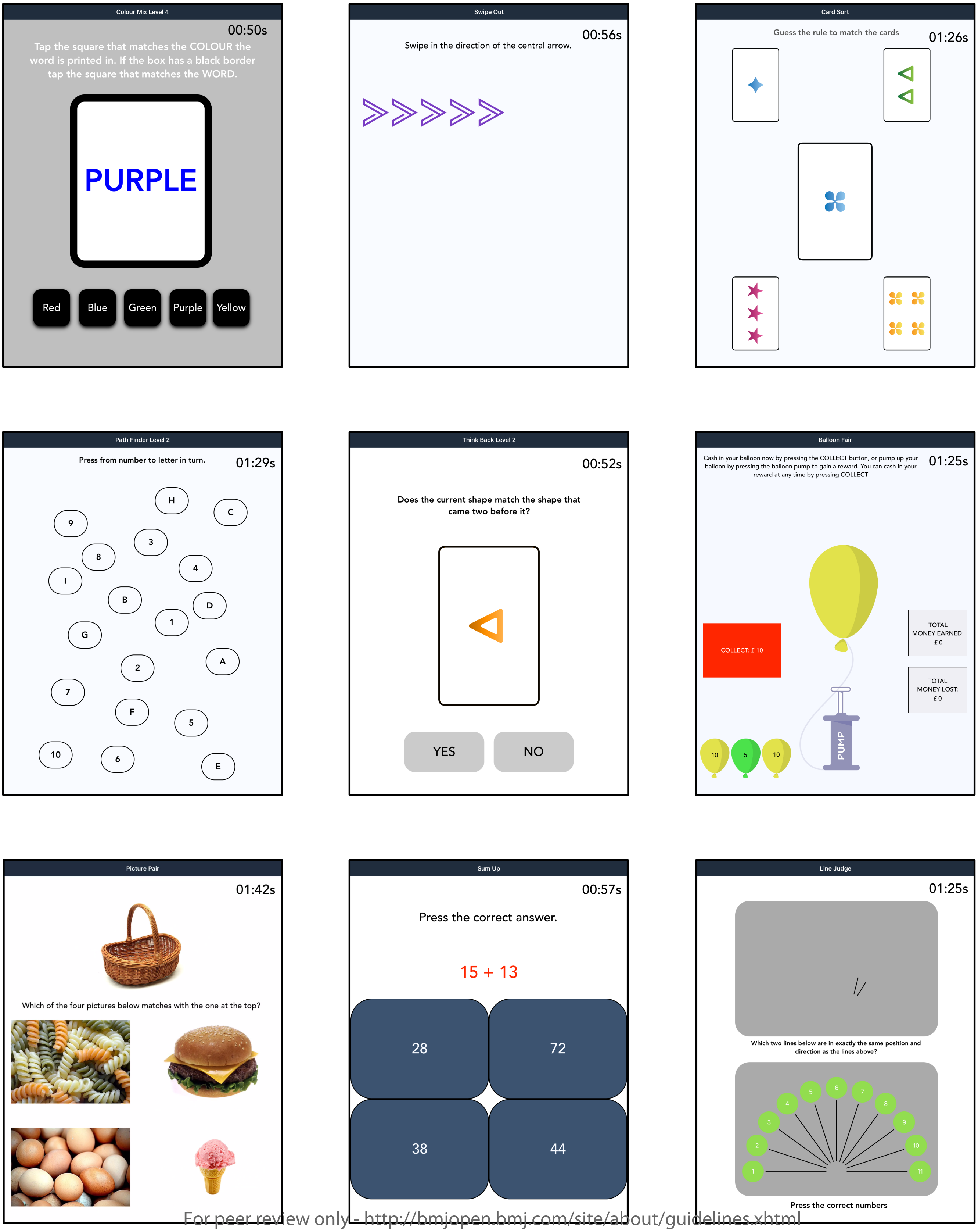

For peer review only - http://bmjopen.bmj.com/site/about/guidelines.xhtml

BMJ Open

Ignite – a novel computerized cognitive assessment battery for frontotemporal dementia

Journal:	*BMJ Open*
Manuscript ID	bmjopen-2021-055211.R1
Article Type:	Protocol
Date Submitted by the Author:	26-Mar-2022
Complete List of Authors:	Moore, Katrina M; UCL Queen Square Institute of Neurology, Department of Neurodegenerative Disease<br>Convery, Rhian S.; UCL Queen Square Institute of Neurology, Department of Neurodegenerative Disease<br>Rohrer, Jonathan; UCL Queen Square Institute of Neurology, Department of Neurodegenerative Disease
**Primary Subject Heading**:	Neurology
Secondary Subject Heading:	Genetics and genomics
Keywords:	Dementia < NEUROLOGY, Adult neurology < NEUROLOGY, Delirium & cognitive disorders < PSYCHIATRY

SCHOLARONE™ Manuscripts

STUDY PROTOCOL

Main title

Ignite – a novel computerized cognitive assessment battery for frontotemporal dementia

Authors

Katrina M Moore PhD[1], Rhian S. Convery MSc[1], Jonathan D Rohrer FRCP PhD[1]

Affiliations

[1]Dementia Research Centre, Department of Neurodegenerative Disease, UCL Queen Square Institute of Neurology, London, UK.

Corresponding author

Professor Jonathan Rohrer, Dementia Research Centre, Department of Neurodegenerative Disease, UCL Institute of Neurology, Queen Square, London, WC1N 3BG, j.rohrer@ucl.ac.uk

For peer review only

Abstract

Introduction: The term frontotemporal dementia (FTD) refers to a heterogeneous group of neurodegenerative disorders affecting the frontal and temporal lobes. Cognitively, impairment of executive function and social cognition predominate across the FTD spectrum although other domains can be affected. Traditionally, cognition is tested through standard 'pen and paper' tasks in FTD. However, recent attempts have been made across other neurodegenerative disorders such as Alzheimer's disease to develop computerized batteries that allow more accurate and sensitive detection of cognitive impairment.

Methods: In this paper we describe the development of Ignite, a novel battery of tests for a tablet computer, particularly focused on FTD. It consists of twelve different tasks which aim to tap into information processing speed, various aspects of executive function, social cognition, semantic knowledge, calculation and visuospatial skills.

Discussion Future studies will focus on validating the battery in a healthy control cohort, comparing it against a standard 'pen and paper' psychometric battery, and finally testing it within an FTD cohort, including those with genetic forms of FTD where we will be able to assess its ability to detect very early cognitive deficits prior to the onset of symptoms.

Keywords: frontotemporal dementia, progranulin, tau, C9orf72, cognition

For peer review only

STRENGTHS AND LIMITATIONS

- This is the first computerized cognitive battery to focus specifically on FTD.
- Tests are short and can be performed at home, reducing participant burden within studies.
- The study will require validation including comparison with a gold standard 'pen and paper' psychometric battery before it can be used more widely.

For peer review only

INTRODUCTION

Frontotemporal dementia (FTD) is a clinically, pathologically and genetically diverse neurodegenerative disease.[1] It is probably the commonest form of dementia in those under 60 years of age and is associated with two main clinical presentations: an impairment in personality and social conduct, known as behavioural variant FTD (bvFTD), and deficits in speech and language, called primary progressive aphasia (PPA). Around a third of FTD is familial,[2] with the majority of autosomal dominant FTD linked to mutations in three key genes: chromosome 9 open reading frame 72 (*C9orf72*), progranulin (*GRN*) and microtubule-associated protein tau (*MAPT*).

The cognitive deficits found in FTD are commonly in the executive function and social cognition domains for bvFTD,[3-5] and the language and semantic knowledge domains for those with PPA, [5-8] although there is often overlap between these disorders. Classically, episodic memory and more posterior cortical functions are spared, particularly in the early stages of the disease.[9] The trajectory of cognitive impairment has now been studied across a number of cohorts, with the earliest change being studied mostly within genetic cohorts where at-risk individuals who are many years from expected symptom onset can be investigated. Deficits across several cognitive tasks have been shown around five to eight years prior to symptom onset particularly in tests of executive function and social cognition, although the pattern of deficits can differ according to which mutation is carried.[10-16] However, prior studies have been performed mainly using traditional 'pen and paper' tasks which may well be limited in their ability to detect subtle changes. Using magnetic resonance imaging, neuroanatomical changes can be seen in presymptomatic genetic FTD studies much earlier, potentially over 20 years prior to expected symptom onset,[12] and so there is a need to bridge the gap between the sensitivity of neuroimaging techniques and current cognitive assessments.

For peer review only

Treatment trials in FTD are currently underway, and although many of the planned studies are focused on symptomatic FTD, it is likely that future studies will also include people in the presymptomatic phase of the disease. One of the greatest challenges facing trials of this nature is having robust cognitive outcome measures. The Food and Drug Administration have recognized that current assessments are not optimal, highlighting the need for improved cognitive markers as outcome measures for clinical trials,[17] and have encouraged the development of novel approaches to evaluate subtle deficits that may emerge in the presymptomatic stages of dementia.[18] Here we describe the design and protocol of Ignite, a novel computerized cognitive assessment tool designed for FTD. We outline the key objectives for developing a computerized battery and provide an overview of the cognitive domains tested within the battery.

METHODS

Patient and Public Involvement

It is standard for traditional cognitive assessments to be performed by trained psychologists in hospital or research settings, which can be both time consuming and costly. On discussion with research participants they describe frequently experiencing high levels of anxiety in relation to attending research visits, particularly in relation to neuropsychological asssessment. In presymptomatic FTD studies this can be most prominent in those who have yet to find out their carrier status. In addition, the number of in person contact days required for a clinical trial can be burdensome and can prevent an individual's participation. With these considerations in mind, we wanted to design a computerized battery that could be performed by individuals themselves, with the aim of reducing participant anxiety, thus ensuring that their best performance is captured. We therefore initially discussed the development of this battery with a small group of people at-risk of familial FTD to ensure we could maximize the user-friendliness of the battery and reduce the participant burden.

For peer review only

Objectives for developing a computerized cognitive battery

Apart from improving the participant experience, we had a number of other objectives for developing the battery:

Comprehensiveness

We wanted to develop a set of assessments that could capture an individual's performance across a number of cognitive domains in a comprehensive manner for FTD. A major drawback of current cognitive assessments is the duration of testing required to get an accurate picture of individual performance, often taking a number of hours. The new battery was designed to be completed in around 30 minutes or less.

Increased sensitivity

Ignite consists mainly of tests which are modifications of existing cognitive tasks. However, each task was modified to shorten the time allowed for completion, with the measurement of reaction times aiming to increase the sensitivity to detect deficits.

Self-assessment

A key aim in the development of Ignite was its ability to be performed by individuals on their own, ideally while at home. All assessments were designed to be as intuitive as possible, appearing more like short games than standard cognitive tests, including the use of simple on-screen buttons or swipes to record answers. A challenge in designing tasks with self-assessment in mind is ensuring that participants are motivated to complete the tasks on their own and that they understand what is being asked of them. To assist participants in understanding and remembering task instructions, they are provided with brief written instructions prior to beginning each individual assessment task. Accompanying the written

For peer review only

instructions are short videos to ensure participants can clearly visualize and understand what is required of them. In addition, once a task has begun, a prompt of the task instruction is displayed at the top of the screen for the duration of the task. In this way, participants can refresh their memories of the task instructions at any time without having to exit the task. To keep participants motivated and engaged each task was designed to range from 30-180 seconds in duration, with most tasks programmed to time out after 60 seconds.

For peer review only

PROTOCOL

Ignite consists of twelve separate assessments that measure a spectrum of cognitive functions (Table 1, Figure 1). Each test was included based on knowledge of the deficits seen in FTD, and in particular those domains most likely to be affected presymptomatically. The tests are self-administered in a predetermined order, in an environment familiar to the participant, such as their home. At present, the app is available on the iPad via the App Store, although future iterations will aim to make versions available for other tablets and operating systems.

Executive function

The term executive function describes a number of different aspects of cognition including reasoning, problem solving and planning [19] commonly related to the workings of the frontal lobe. It consists of a number of different subdomains, which include inhibitory control, cognitive flexibility and working memory.[20] We aimed to design tasks tapping into each of these subdomains as well as decision making and cognitive timing as previous studies of FTD have shown deficits within all of them,[21-23] and in particular impairment can be seen presymptomatically across each of the major genetic groups.[12,24]

For peer review only - http://bmjopen.bmj.com/site/about/guidelines.xhtml

Inhibitory control

This subdomain is described as the ability to control one's attention, behaviour and thoughts to override a strong internal predisposition or external lure.[20] It is abnormal in FTD and relates to the key symptom of disinhibition that forms part of the diagnostic criteria for behavioural variant FTD.[25] We included two tasks of inhibitory control.

Colour Mix

In our task, we adapted the classical Stroop task including five different colours: red, blue, green, purple and yellow. The initial two levels of the task test speed of processing. In Level 1, participants are presented with a coloured circle and all five colour names written below. They are asked to match the colour of the circle with the correct colour name from 5. They have 30 seconds to complete as many as possible (with a maximum of 50 possible trials). In Level 2, participants are presented with the name of a colour and (as with Level 1) the five colour names written below. They are asked to match the target colour word with the matching word below. They have 30 seconds to complete as many as possible (with a maximum of 50 possible trials). The next two levels measure inhibitory control. In Level 3, participants are presented with a colour word written in a different colour e.g. BLUE written in red ink. They are asked to match the colour of the ink (rather than the name) to one of the five colour names written below. They have 60 seconds to complete as many as possible (with a maximum of 50 possible trials). Lastly, in Level 4, participants are presented as in Level 3 with a colour word written in a different colour, and have to complete the task as in Level 3, unless a black border appears around the word (as it does on some trials), when they have to match the written word rather than the ink colour (e.g. match blue if the word is BLUE written in red ink) to one of the five colour names written below. They have 60 seconds to complete as many as possible (with a maximum of 50 possible trials).

For peer review only

Swipe Out

Our task was a modification of the classical Flanker task, with an arrowhead (facing up, down, left or right) with four flanking arrowheads surrounding the target arrowhead. Participants are asked to swipe in the direction of the central arrowhead. In some trials the flanking arrowheads are facing in the same direction, and in some trials they are facing in the opposite direction. Participants have 60 seconds to complete as many trials as possible (with a maximum of 40 possible trials).

Cognitive flexibility

This subdomain describes the ability to change perspectives from an established pattern, and mentally adjust or 'set shift' our perspective to new task demands. Tasks that tap into cognitive flexibility include the Wisconsin Card Sorting Task (WCST) [26] and the Trail Making Test (TMT) [27]. Deficits in both of these tasks have been previously described in FTD [10,13,28] including presymptomatically in those with genetic forms of FTD.[29]

Card Sort

Our task adapts a classical card sorting task, asking participants to sort cards by dragging them from the centre of the screen into card deposits located in the four corners of the screen. Participants are required to sort cards by a rule: either shape, number or colour. The primary aim of the task is for the participant to understand the correct sorting criteria on the basis of feedback, either correct or incorrect, and to switch flexibly between sorting rules whenever feedback indicates that the rule has changed. After a set number of correct card sorts are performed the rule changes, and negative feedback is given if participants continue to sort by the previous rule. The successful performance of the task is measured in participants' ability to flexibly respond to feedback given in shifting to the new rule.[26] The number of perseverative errors, identified by participants failing to adapt to a new rule, is thought to be indicative of poor cognitive

For peer review only

flexibility.[30] In our task, participants have a total of 90 seconds in order to sort as many as cards as possible.

Path Finder

This task adapts the Trail Making Test (TMT), asking participants to tap stimuli in sequential order (rather than draw an unbroken line between letters and/or numbers as in the original pen and paper task). Correct sequencing is indicated by the outline of each letter or number changing colour to green. The letter or number changes to red if selected incorrectly. In Level 1 participants are asked to select numbers in the correct order (similar to TMT part A), with a maximum of 60 seconds to complete the task. In Level 2 participants are asked to alternate between numbers and letters in the correct order (similar to TMT part B), with a maximum of 90 seconds to complete the task.

Working memory

This subdomain of executive function describes the ability to hold information in mind and mentally work with it.[31] A classic paradigm for assessing working memory is the N-back task, which requires participants to monitor a series of stimuli and respond whenever the stimuli presented matches the properties of the trial presented 'N' before it.[32] The task requires the monitoring, updating and manipulation of information displayed to perform the task effectively.

Think Back

There are two levels to our N-Back task. Level 1 is a 1-back task and Level 2 is a 2-back task. In Level 1 participants must indicate whether a shape matches the one preceding it. In Level 2 participants must indicate whether a shape matches the shape shown two before it, intended to place greater demand on

For peer review only

working memory. An answer is submitted by pressing a ‘yes’ or ‘no’ button. In each level, participants have 60 seconds to complete as many trials as possible (up to a maximum of 72 trials).

Decision making

For many people with FTD their ability to make decisions is compromised.[33] Several paradigms are employed in assessing decision making behaviour, often in the form of gambling or risk-taking tasks, such as the Iowa Gambling Task,[34] Cambridge Gambling Task,[35] Game of Dice Task [36] and the Balloon Analogue Risk Task,[37] with people with FTD shown to be impaired on these tests.[38]

Balloon Fair

In our task we adapted the Balloon Analogue Risk Task [37] to assess decision making behaviour. Participants are presented with a simulated balloon and balloon pump. Also presented is a button labelled ‘Collect £’ and a permanent money counter labelled ‘Total Earned’, alongside a counter displaying money that has been lost. With each press on the pump, the balloon is inflated, and money is accumulated in a temporary bank. Balloons pop after a predetermined ‘popping’ point, at which point the money accrued in the temporary bank is lost. Participants have the option to cash in the money accumulated in the temporary bank at any point by clicking the ‘Collect £’ button. After each balloon has popped (money lost) or the money has been collected a new balloon appears. Balloons are differently coloured (green, yellow and blue) with each colour associated with a unique sum (£5, £10, £50 respectively), and a unique popping point (quicker for higher sums). Participants are unaware of the probability of a balloon popping. The aim of the task is to earn as much money as possible. Participants have 90 seconds to complete the task.

Cognitive timing

For peer review only

The concept of timing is integral to performing many mental processes.[39] Although not well-studied in FTD, one previous study has shown impairment in both externally-paced and self-paced finger tapping.[23]

Time Tap

We adapted the task described in Henley et al., 2014 [23] to test cognitive timing. A pulsating circle is presented in the middle of the top of the screen and a tone sounds in time to the pulse (at 1500ms intervals). Participants are asked to use the index finger of their dominant hand to tap in time with the pulsating circle on a second circle presented below. After 30 seconds the pulsation and tone cease, and participants are instructed to maintain the same tempo for a further 30 seconds.

Social Cognition

Social cognition is the ability to perceive, interpret and generate a response to the intentions, behaviours, and feelings of others, and includes a number of subdomains such as emotion recognition and theory of mind. Social cognition impairment occurs early and almost universally in all people with FTD [40,41] as well as those with other forms of FTD, such as PPA.[42,43] These deficits include poor recognition of simple emotions in faces,[44,45] as well as impairment of complex emotion processing, and other tasks of theory of mind [45,46] Early deficits have been seen in genetic FTD including those who are presymptomatic.[13]

Face Match

In our task of simple emotion processing participants are presented with a target basic emotion word (sadness, happiness, anger, fear, disgust, surprise) at the top of the screen alongside nine images of faces in the corners of the screen.[47] Participants are asked to tap all of the faces displaying the target emotion as quickly as possible. There are five correct answers out of each nine presented faces, and participants

For peer review only

must press all five within 10 seconds, or the task moves on to the next set of nine faces. The participants have to complete as many items as possible within 60 seconds (up to a maximum of 6 sets of nine faces).

Mind Reading

Our task of complex emotion processing adapts the Reading the Mind in the Eyes test so that participants are presented with a target emotion word in the centre of the screen alongside four images of eyes in the corners of the screen, one of which matches the emotion. Participants are asked to select the image which best matches the target emotion word, completing as many as possible within 90 seconds (up to a maximum of 20 words).

Semantic knowledge

Impairment of semantic knowledge is characteristic of the semantic variant of primary progressive aphasia but is seen in other forms of FTD, and has been shown to occur presymptomatically, particularly in those with *MAPT* mutations.[48,49]

Picture Pair

Our task uses stimuli from the modified version of the Camel and Cactus task that we have previously described as detecting early semantic deficits in genetic FTD.[50] The participant has to match a target picture with a semantically-linked matching picture from a choice of four e.g. matching Camel with Cactus rather than a tree, sunflower or rose. The participants have to answer as many questions as possible in 120 seconds (with a maximum of 25 questions).

Arithmetic

For peer review only

Calculation abilities have been shown to be highly dependent on left parietal lobe function.[51,52] Although initial studies focused on anterior involvement of the frontal and temporal lobes in FTD, more recent studies have shown that some forms of FTD can have more posterior cortical atrophy, particularly those with *GRN* mutations and *C9orf72* expansions,[53] including presymptomatically.[12] Dyscalculia has been reported in both of these groups.[54,55] Although deficits are generally not limited to one form of mental arithmetic, one study has shown deficits specifically in multiplication in ALS.[56]

Sum Up

Our task includes additions, subtractions, multiplications and divisions, with participants presented with the sum and four possible answers, and a time limit of 10 seconds for completion of each sum. The participants have to answer as many questions as possible within 60 seconds (with a maximum of 48 sums, 12 of each type).

Visuospatial skills

Visuospatial processing is classically associated with right parietal lobe function.[57] As mentioned above, more posterior cortical atrophy, including involvement of the right parietal lobe has been seen in genetic FTD. Given this finding, it is unsurprising that several studies have highlighted impairments of visuospatial skills in both *GRN* mutation carriers [58,59] and *C9orf72* expansion carriers.[60]

Line Judge

Judgement of line orientation is a standard measure of visuospatial skills,[61,62] testing an individual's ability to match the angle and orientation of lines in space, with deficits seen in forms of dementia such as Alzheimer's diseases and Dementia with Lewy Bodies which are usually associated with parietal atrophy.[63,64]

For peer review only

In our task, participants are presented with pairs of angled lines and are asked to match the target pair of lines to a set of 11 lines arranged in a semicircle and numbered 1 to 11. Participants press the correct numbers that correspond to the line pair. The participants have to answer as many questions as possible within a 90 second timeframe (up to a maximum of 15 pairs).

Discussion

Ignite is a novel computerized battery of assessments for the detection of cognitive changes in FTD. It includes tasks of information processing speed, executive function, social cognition, semantic knowledge, arithmetic and visuospatial skills. It potentially may be sensitive enough to detect early (and presymptomatic) deficits within an at-risk genetic cohort. Ignite will be initially validated in a healthy control cohort and compared against a standard set of 'pen and paper' psychometric tasks, including assessment of test-retest reliability. The battery will then be tested within an FTD cohort such as GENFI (www.genfi.org; [12]) where it would be possible to assess both presymptomatic and symptomatic people with FTD. In the future we would also hope to be able to compare Ignite with other emerging computerized batteries aimed specifically at FTD, such as those being developed as part of the ARTFL-LEFFTDS Longitudinal Frontotemporal Lobar Degeneration (ALLFTD*:* www.allftd.org*)* study.

Conclusions

The final goal of the Ignite study will be to develop a set of validated tasks which are able to track disease progression in FTD, specifically in the presymptomatic and very early symptomatic phase of the disease, and therefore have the potential to be employed in clinical trials as a cognitive outcome measure.

Abbreviations

For peer review only

FTD, frontotemporal dementia; PPA, primary progressive aphasia; C9orf72, chromosome 9 open reading frame 72; GRN, progranulin; MAPT, microtubule-associated protein tau; bvFTD, behavioural variant frontotemporal dementia, TMT, Trail Making Test; WCST, Wisconsin Card Sorting Task.

For peer review only

Declarations

Ethics approval and consent to participate

The Ignite study has been approved by the UCL ethics committee. Written informed consent is being obtained for those taking part in the study.

Consent to publish

Not applicable

Availability of data and materials

The Ignite app is free to download and use from the Appstore.

Competing interests

KMM is now an employee of Ionis Pharmaceuticals. JDR has served on medical advisory boards or provided consultancy for Alector, Arkuda Therapeutics, Wave Life Sciences, Prevail Therapeutics, UCB, AC Immune, Astex Pharmaceuticals, Biogen, Takeda and Eisai.

Funding

The app was developed initially through an Alzheimer's Society PhD studentship (for KMM) awarded to JDR (AS-PhD-2015-005). RSC has been funded by a Frontotemporal Dementia Research Studentship in Memory of David Blechner awarded through The National Brain Appeal (RCN 290173). JDR has received funding from an MRC Clinician Scientist Fellowship (MR/M008525/1), an NIHR Rare Disease Translational Research Collaboration (BRC149/NS/MH) and the Brain Research UK Miriam Marks Senior Fellowship; his work is also supported by the MRC UK GENFI grant (MR/M023664/1), the Bluefield Project and the JPND GENFI-PROX grant (2019-02248). None of the funding bodies had any direct input into the app development.

Authors' contributions

KMM, RSC and JDR wrote the manuscript. All authors have read and approved the manuscript.

Acknowledgements

We thank the research participants at the Dementia Research Centre for their contribution to the development of Ignite.

For peer review only

References

1. Warren, J. D., Rohrer, J. D., & Rossor, M. N. (2013). Clinical review. Frontotemporal dementia. BMJ (Clinical research ed.), 347, f4827.

2. Rohrer, J. D., Guerreiro, R., Vandrovcova, J., Uphill, J., Reiman, D., Beck, J., Isaacs, A. M., Authier, A., Ferrari, R., Fox, N. C., Mackenzie, I. R., Warren, J. D., de Silva, R., Holton, J., Revesz, T., Hardy, J., Mead, S., & Rossor, M. N. (2009). The heritability and genetics of frontotemporal lobar degeneration. Neurology, 73(18), 1451–1456.

3. Grossman M. (2002). Frontotemporal dementia: a review. Journal of the International Neuropsychological Society : JINS, 8(4), 566–583.

4. Harciarek, M., & Jodzio, K. (2005). Neuropsychological differences between frontotemporal dementia and Alzheimer's disease: a review. Neuropsychology review, 15(3), 131–145.

5. Harciarek, M., & Cosentino, S. (2013). Language, executive function and social cognition in the diagnosis of frontotemporal dementia syndromes. International review of psychiatry (Abingdon, England), 25(2), 178–196.

6. Warren, J. D., Rohrer, J. D., & Rossor, M. N. (2013). Clinical review. Frontotemporal dementia. BMJ (Clinical research ed.), 347, f4827.

7. Woollacott, I. O., & Rohrer, J. D. (2016). The clinical spectrum of sporadic and familial forms of frontotemporal dementia. Journal of neurochemistry, 138 Suppl 1, 6–31.

8. Deleon, J., & Miller, B. L. (2018). Frontotemporal dementia. Handbook of clinical neurology, 148, 409–430.

9. Gorno-Tempini, M. L., Hillis, A. E., Weintraub, S., Kertesz, A., Mendez, M., Cappa, S. F., Ogar, J. M., Rohrer, J. D., Black, S., Boeve, B. F., Manes, F., Dronkers, N. F., Vandenberghe, R., Rascovsky, K., Patterson, K., Miller, B. L., Knopman, D. S., Hodges, J. R., Mesulam, M. M., & Grossman, M.

For peer review only

(2011). Classification of primary progressive aphasia and its variants. Neurology, 76(11), 1006–1014.

10. Geschwind, D. H., Robidoux, J., Alarcón, M., Miller, B. L., Wilhelmsen, K. C., Cummings, J. L., & Nasreddine, Z. S. (2001). Dementia and neurodevelopmental predisposition: cognitive dysfunction in presymptomatic subjects precedes dementia by decades in frontotemporal dementia. Annals of neurology, 50(6), 741–746.
11. Hallam, B. J., Jacova, C., Hsiung, G. Y., Wittenberg, D., Sengdy, P., Bouchard-Kerr, P., Slack, P., Rademakers, R., Baker, M., Chow, T. W., Levine, B., Feldman, H. H., & Mackenzie, I. R. (2014). Early neuropsychological characteristics of progranulin mutation carriers. Journal of the International Neuropsychological Society : JINS, 20(7), 694–703.
12. Rohrer, J. D., Nicholas, J. M., Cash, D. M., van Swieten, J., Dopper, E., Jiskoot, L., van Minkelen, R., Rombouts, S. A., Cardoso, M. J., Clegg, S., Espak, M., Mead, S., Thomas, D. L., De Vita, E., Masellis, M., Black, S. E., Freedman, M., Keren, R., MacIntosh, B. J., Rogaeva, E., … Binetti, G. (2015). Presymptomatic cognitive and neuroanatomical changes in genetic frontotemporal dementia in the Genetic Frontotemporal dementia Initiative (GENFI) study: a cross-sectional analysis. The Lancet. Neurology, 14(3), 253–262.
13. Jiskoot, L. C., Dopper, E. G., Heijer, T. d., Timman, R., van Minkelen, R., van Swieten, J. C., & Papma, J. M. (2016). Presymptomatic cognitive decline in familial frontotemporal dementia: A longitudinal study. Neurology, 87(4), 384–391.
14. Papma, J. M., Jiskoot, L. C., Panman, J. L., Dopper, E. G., den Heijer, T., Donker Kaat, L., Pijnenburg, Y., Meeter, L. H., van Minkelen, R., Rombouts, S., & van Swieten, J. C. (2017). Cognition and gray and white matter characteristics of presymptomatic C9orf72 repeat expansion. Neurology, 89(12), 1256–1264.

For peer review only

15. Suhonen, N. M., Haanpää, R. M., Korhonen, V., Jokelainen, J., Pitkäniemi, A., Heikkinen, A. L., Krüger, J., Hartikainen, P., Helisalmi, S., Hiltunen, M., Hänninen, T., & Remes, A. M. (2017). Neuropsychological Profile in the C9ORF72 Associated Behavioral Variant Frontotemporal Dementia. Journal of Alzheimer's disease : JAD, 58(2), 479–489.

16. Jiskoot, L. C., Panman, J. L., van Asseldonk, L., Franzen, S., Meeter, L., Donker Kaat, L., van der Ende, E. L., Dopper, E., Timman, R., van Minkelen, R., van Swieten, J. C., van den Berg, E., & Papma, J. M. (2018). Longitudinal cognitive biomarkers predicting symptom onset in presymptomatic frontotemporal dementia. Journal of neurology, 265(6), 1381–1392.

17. Sabbagh, M. N., Hendrix, S., & Harrison, J. E. (2019). FDA position statement "Early Alzheimer's disease: Developing drugs for treatment, Guidance for Industry". Alzheimer's & dementia (New York, N. Y.), 5, 13–19.

18. FDA (2018). Early Alzheimer’s Disease: Developing Drugs for Treatment Guidance for Industry DRAFT GUIDANCE. Retrieved from https://www.fda.gov/Drugs/GuidanceComplianceRegulatoryInformation/Guidances/default.htm

19. Collins, A., & Koechlin, E. (2012). Reasoning, learning, and creativity: frontal lobe function and human decision-making. PLoS biology, 10(3), e1001293.

20. Diamond A. (2013). Executive functions. Annual review of psychology, 64, 135–168.

21. Strenziok, M., Pulaski, S., Krueger, F., Zamboni, G., Clawson, D., & Grafman, J. (2011). Regional brain atrophy and impaired decision making on the balloon analog risk task in behavioral variant frontotemporal dementia. Cognitive and behavioral neurology : official journal of the Society for Behavioral and Cognitive Neurology, 24(2), 59–67.

22. Manes, F., Torralva, T., Ibáñez, A., Roca, M., Bekinschtein, T., & Gleichgerrcht, E. (2011). Decision-making in frontotemporal dementia: clinical, theoretical and legal implications. Dementia and geriatric cognitive disorders, 32(1), 11–17.

For peer review only

23. Henley, S. M., Downey, L. E., Nicholas, J. M., Kinnunen, K. M., Golden, H. L., Buckley, A., Mahoney, C. J., & Crutch, S. J. (2014). Degradation of cognitive timing mechanisms in behavioural variant frontotemporal dementia. Neuropsychologia, 65, 88–101.

24. Staffaroni, A. M., Bajorek, L., Casaletto, K. B., Cobigo, Y., Goh, S. M., Wolf, A., Heuer, H. W., Elahi, F. M., Ljubenkov, P. A., Dever, R., Kornak, J., Appleby, B., Bove, J., Bordelon, Y., Brannelly, P., Brushaber, D., Caso, C., Coppola, G., Dheel, C., Dickerson, B. C., … ARTFL/LEFFTDS consortium (2020). Assessment of executive function declines in presymptomatic and mildly symptomatic familial frontotemporal dementia: NIH-EXAMINER as a potential clinical trial endpoint. Alzheimer's & dementia : the journal of the Alzheimer's Association, 16(1), 11–21.

25. Rascovsky, K., Hodges, J. R., Knopman, D., Mendez, M. F., Kramer, J. H., Neuhaus, J., van Swieten, J. C., Seelaar, H., Dopper, E. G., Onyike, C. U., Hillis, A. E., Josephs, K. A., Boeve, B. F., Kertesz, A., Seeley, W. W., Rankin, K. P., Johnson, J. K., Gorno-Tempini, M. L., Rosen, H., Prioleau-Latham, C. E., … Miller, B. L. (2011). Sensitivity of revised diagnostic criteria for the behavioural variant of frontotemporal dementia. Brain : a journal of neurology, 134(Pt 9), 2456–2477.

26. Stuss, D. T., Levine, B., Alexander, M. P., Hong, J., Palumbo, C., Hamer, L., Murphy, K. J., & Izukawa, D. (2000). Wisconsin Card Sorting Test performance in patients with focal frontal and posterior brain damage: effects of lesion location and test structure on separable cognitive processes. Neuropsychologia, 38(4), 388–402.

27. Delis, D. ., Kaplan, E., & J.H, K. (2001). The Delis-Kaplan Executive Function System: Examiner’s Manual. San Andonio: The Psychological Corporation.

28. Rohrer, J. D., Warren, J. D., Omar, R., Mead, S., Beck, J., Revesz, T., Holton, J., Stevens, J. M., Al-Sarraj, S., Pickering-Brown, S. M., Hardy, J., Fox, N. C., Collinge, J., Warrington, E. K., & Rossor, M. N. (2008). Parietal lobe deficits in frontotemporal lobar degeneration caused by a mutation in the progranulin gene. Archives of neurology, 65(4), 506–513.

For peer review only

29. Barandiaran, M., Estanga, A., Moreno, F., Indakoetxea, B., Alzualde, A., Balluerka, N., Martí Massó, J. F., & López de Munain, A. (2012). Neuropsychological features of asymptomatic c.709-1G>A progranulin mutation carriers. Journal of the International Neuropsychological Society : JINS, 18(6), 1086–1090.

30. Lange, F., Brückner, C., Knebel, A., Seer, C., & Kopp, B. (2018). Executive dysfunction in Parkinson's disease: A meta-analysis on the Wisconsin Card Sorting Test literature. Neuroscience and biobehavioral reviews, 93, 38–56.

31. Baddeley A.D, & Hitch G.J. (1994). Developments in the Concept of Working Memory. Neuropsychology, 8, 485–493.

32. Owen, A. M., McMillan, K. M., Laird, A. R., & Bullmore, E. (2005). N-back working memory paradigm: a meta-analysis of normative functional neuroimaging studies. Human brain mapping, 25(1), 46–59.

33. Gleichgerrcht, E., Torralva, T., Roca, M., Szenkman, D., Ibanez, A., Richly, P., Pose, M., & Manes, F. (2012). Decision making cognition in primary progressive aphasia. Behavioural neurology, 25(1), 45–52.

34. Bechara, A., & Van Der Linden, M. (2005). Decision-making and impulse control after frontal lobe injuries. Current opinion in neurology, 18(6), 734–739.

35. Rogers, R. D., Owen, A. M., Middleton, H. C., Williams, E. J., Pickard, J. D., Sahakian, B. J., & Robbins, T. W. (1999). Choosing between small, likely rewards and large, unlikely rewards activates inferior and orbital prefrontal cortex. The Journal of neuroscience : the official journal of the Society for Neuroscience, 19(20), 9029–9038.

36. Brand, M., Fujiwara, E., Borsutzky, S., Kalbe, E., Kessler, J., & Markowitsch, H. J. (2005). Decision-making deficits of korsakoff patients in a new gambling task with explicit rules: associations with executive functions. Neuropsychology, 19(3), 267–277.

For peer review only

37. Lejuez, C. W., Read, J. P., Kahler, C. W., Richards, J. B., Ramsey, S. E., Stuart, G. L., Strong, D. R., & Brown, R. A. (2002). Evaluation of a behavioral measure of risk taking: the Balloon Analogue Risk Task (BART). Journal of experimental psychology. Applied, 8(2), 75–84.

38. Rahman, S., Sahakian, B. J., Hodges, J. R., Rogers, R. D., & Robbins, T. W. (1999). Specific cognitive deficits in mild frontal variant frontotemporal dementia. Brain : a journal of neurology, 122 (Pt 8), 1469–1493.

39. Allman, M. J., & Meck, W. H. (2012). Pathophysiological distortions in time perception and timed performance. Brain : a journal of neurology, 135(Pt 3), 656–677.

40. Snowden, J. S., Gibbons, Z. C., Blackshaw, A., Doubleday, E., Thompson, J., Craufurd, D., Foster, J., Happé, F., & Neary, D. (2003). Social cognition in frontotemporal dementia and Huntington's disease. Neuropsychologia, 41(6), 688–701.

41. Russell, L. L., Greaves, C. V., Bocchetta, M., Nicholas, J., Convery, R. S., Moore, K., Cash, D. M., van Swieten, J., Jiskoot, L., Moreno, F., Sanchez-Valle, R., Borroni, B., Laforce, R., Jr, Masellis, M., Tartaglia, M. C., Graff, C., Rotondo, E., Galimberti, D., Rowe, J. B., Finger, E., … Genetic FTD Initiative, GENFI (2020). Social cognition impairment in genetic frontotemporal dementia within the GENFI cohort. Cortex; a journal devoted to the study of the nervous system and behavior, 133, 384–398.

42. Couto, B., Manes, F., Montañés, P., Matallana, D., Reyes, P., Velasquez, M., Yoris, A., Baez, S., & Ibáñez, A. (2013). Structural neuroimaging of social cognition in progressive non-fluent aphasia and behavioral variant of frontotemporal dementia. Frontiers in human neuroscience, 7, 467.

43. Fittipaldi, S., Ibanez, A., Baez, S., Manes, F., Sedeno, L., & Garcia, A. M. (2019). More than words: Social cognition across variants of primary progressive aphasia. Neuroscience and biobehavioral reviews, 100, 263–284.

For peer review only

44. Kumfor, F., & Piguet, O. (2012). Disturbance of emotion processing in frontotemporal dementia: a synthesis of cognitive and neuroimaging findings. Neuropsychology review, 22(3), 280–297.

45. Lough, S., Kipps, C. M., Treise, C., Watson, P., Blair, J. R., & Hodges, J. R. (2006). Social reasoning, emotion and empathy in frontotemporal dementia. Neuropsychologia, 44(6), 950–958.

46. Gregory, C., Lough, S., Stone, V., Erzinclioglu, S., Martin, L., Baron-Cohen, S., & Hodges, J. R. (2002). Theory of mind in patients with frontal variant frontotemporal dementia and Alzheimer's disease: theoretical and practical implications. Brain : a journal of neurology, 125(Pt 4), 752–764.

47. Tottenham, N., Tanaka, J. W., Leon, A. C., McCarry, T., Nurse, M., Hare, T. A., Marcus, D. J., Westerlund, A., Casey, B. J., & Nelson, C. (2009). The NimStim set of facial expressions: judgments from untrained research participants. Psychiatry research, 168(3), 242–249.

48. Snowden, J. S., Adams, J., Harris, J., Thompson, J. C., Rollinson, S., Richardson, A., Jones, M., Neary, D., Mann, D. M., & Pickering-Brown, S. (2015). Distinct clinical and pathological phenotypes in frontotemporal dementia associated with MAPT, PGRN and C9orf72 mutations. Amyotrophic lateral sclerosis & frontotemporal degeneration, 16(7-8), 497–505.

49. Hardy, C. J., Buckley, A. H., Downey, L. E., Lehmann, M., Zimmerer, V. C., Varley, R. A., Crutch, S. J., Rohrer, J. D., Warrington, E. K., & Warren, J. D. (2016). The Language Profile of Behavioral Variant Frontotemporal Dementia. Journal of Alzheimer's disease : JAD, 50(2), 359–371.

50. Moore, K., Convery, R., Bocchetta, M., Neason, M., Cash, D. M., Greaves, C., Russell, L. L., Clarke, M., Peakman, G., van Swieten, J., Jiskoot, L., Moreno, F., Barandiaran, M., Sanchez-Valle, R., Borroni, B., Laforce, R., Jr, Doré, M. C., Masellis, M., Tartaglia, M. C., Graff, C., … Genetic FTD Initiative, GENFI* (2020). A modified Camel and Cactus Test detects presymptomatic semantic impairment in genetic frontotemporal dementia within the GENFI cohort. Applied neuropsychology. Adult, 1–8.

For peer review only

51. Takayama, Y., Sugishita, M., Akiguchi, I., & Kimura, J. (1994). Isolated acalculia due to left parietal lesion. Archives of neurology, 51(3), 286–291.

52. Dehaene, S., Piazza, M., Pinel, P., & Cohen, L. (2003). Three parietal circuits for number processing. Cognitive neuropsychology, 20(3), 487–506.

53. Cash, D. M., Bocchetta, M., Thomas, D. L., Dick, K. M., van Swieten, J. C., Borroni, B., Galimberti, D., Masellis, M., Tartaglia, M. C., Rowe, J. B., Graff, C., Tagliavini, F., Frisoni, G. B., Laforce, R., Jr, Finger, E., de Mendonça, A., Sorbi, S., Rossor, M. N., Ourselin, S., Rohrer, J. D., … Genetic FTD Initiative, GENFI (2018). Patterns of gray matter atrophy in genetic frontotemporal dementia: results from the GENFI study. Neurobiology of aging, 62, 191–196.

54. Rohrer, J. D., Warren, J. D., Barnes, J., Mead, S., Beck, J., Pepple, T., Boyes, R., Omar, R., Collinge, J., Stevens, J. M., Warrington, E. K., Rossor, M. N., & Fox, N. C. (2008). Mapping the progression of progranulin-associated frontotemporal lobar degeneration. Nature clinical practice. Neurology, 4(8), 455–460.

55. Mahoney, C. J., Downey, L. E., Ridgway, G. R., Beck, J., Clegg, S., Blair, M., Finnegan, S., Leung, K. K., Yeatman, T., Golden, H., Mead, S., Rohrer, J. D., Fox, N. C., & Warren, J. D. (2012). Longitudinal neuroimaging and neuropsychological profiles of frontotemporal dementia with C9ORF72 expansions. Alzheimer's research & therapy, 4(5), 41.

56. Palmieri, A., Soraru, G., D'Ascenzo, C., Balestreri, S., Arcara, G., Ermani, M., Pegoraro, E., & Semenza, C. (2013). Specific numerical processing impairment in ALS patients. Amyotrophic lateral sclerosis & frontotemporal degeneration, 14(1), 6–12. https://doi.org/10.3109/17482968.2012.719235

57. Bartolomeo, P., Thiebaut de Schotten, M., & Chica, A. B. (2012). Brain networks of visuospatial attention and their disruption in visual neglect. Frontiers in human neuroscience, 6, 110.

For peer review only

58. Taipa, R., Tuna, A., Damásio, J., Pinto, P. S., Cavaco, S., Pereira, S., Milterberger-Miltenyi, G., Galimberti, D., & Melo-Pires, M. (2012). Clinical, neuropathological, and genetic characteristics of the novel IVS9+1delG GRN mutation in a patient with frontotemporal dementia. Journal of Alzheimer's disease : JAD, 30(1), 83–90.

59. Taghdiri, F., Sato, C., Ghani, M., Moreno, D., Rogaeva, E., & Tartaglia, M. C. (2016). Novel GRN Mutations in Patients with Corticobasal Syndrome. Scientific reports, 6, 22913.

60. Patel, A. N., & Sampson, J. B. (2015). Cognitive Profile of C9orf72 in Frontotemporal Dementia and Amyotrophic Lateral Sclerosis. Current neurology and neuroscience reports, 15(9), 59.

61. Benton, A. L., Hamsher, K., Varney, N. R., & Spreen, O. (1983). Contributions to neuropsychological assessment. New York: Oxford University Press.

62. Qualls, C. E., Bliwise, N. G., & Stringer, A. Y. (2000). Short forms of the Benton judgment of line orientation test: Development and psychometric properties. Archives of Clinical Neuropsychology, 15(2), 159–163.

63. Simard, M., van Reekum, R., & Myran, D. (2003). Visuospatial impairment in dementia with Lewy bodies and Alzheimer's disease: a process analysis approach. International journal of geriatric psychiatry, 18(5), 387–391.

64. Salimi, S., Irish, M., Foxe, D., Hodges, J. R., Piguet, O., & Burrell, J. R. (2017). Can visuospatial measures improve the diagnosis of Alzheimer's disease?. Alzheimer's & dementia (Amsterdam, Netherlands), 10, 66–74.

For peer review only

Figure Legends

Figure 1: Examples of tasks in the Ignite battery: top row left *Colour Mix*, top row middle *Swipe Out*, top row right *Card Sort*, middle row left *Path Finder*, middle row middle *Think Back*, middle row right *Balloon Fair*, bottom row left *Picture Pair*, bottom row middle *Sum Up*, bottom row right *Line Judge*.

Table 1: Summary of tasks within the Ignite protocol

<table>
<tr><th></th><th>Cognitive domain</th><th>Cognitive subdomain</th><th>Task</th></tr>
<tr><td>1</td><td rowspan="7">Executive function and information processing speed</td><td rowspan="2">Inhibitory control</td><td>Colour Mix</td></tr>
<tr><td>2</td><td>Swipe Out</td></tr>
<tr><td>3</td><td rowspan="2">Cognitive flexibility</td><td>Card Sort</td></tr>
<tr><td>4</td><td>Path Finder</td></tr>
<tr><td>5</td><td>Working memory</td><td>Think Back</td></tr>
<tr><td>6</td><td>Decision making</td><td>Balloon Fair</td></tr>
<tr><td>7</td><td>Cognitive Timing</td><td>Time Tap</td></tr>
<tr><td>8</td><td rowspan="2">Social cognition</td><td>Emotion processing</td><td>Face Match</td></tr>
<tr><td>9</td><td>Theory of Mind</td><td>Mind Reading</td></tr>
<tr><td>10</td><td colspan="2">Semantic knowledge</td><td>Picture Pair</td></tr>
<tr><td>11</td><td colspan="2">Arithmetic</td><td>Sum Up</td></tr>
<tr><td>12</td><td colspan="2">Visuospatial skills</td><td>Line Judge</td></tr>
</table>

For peer review only

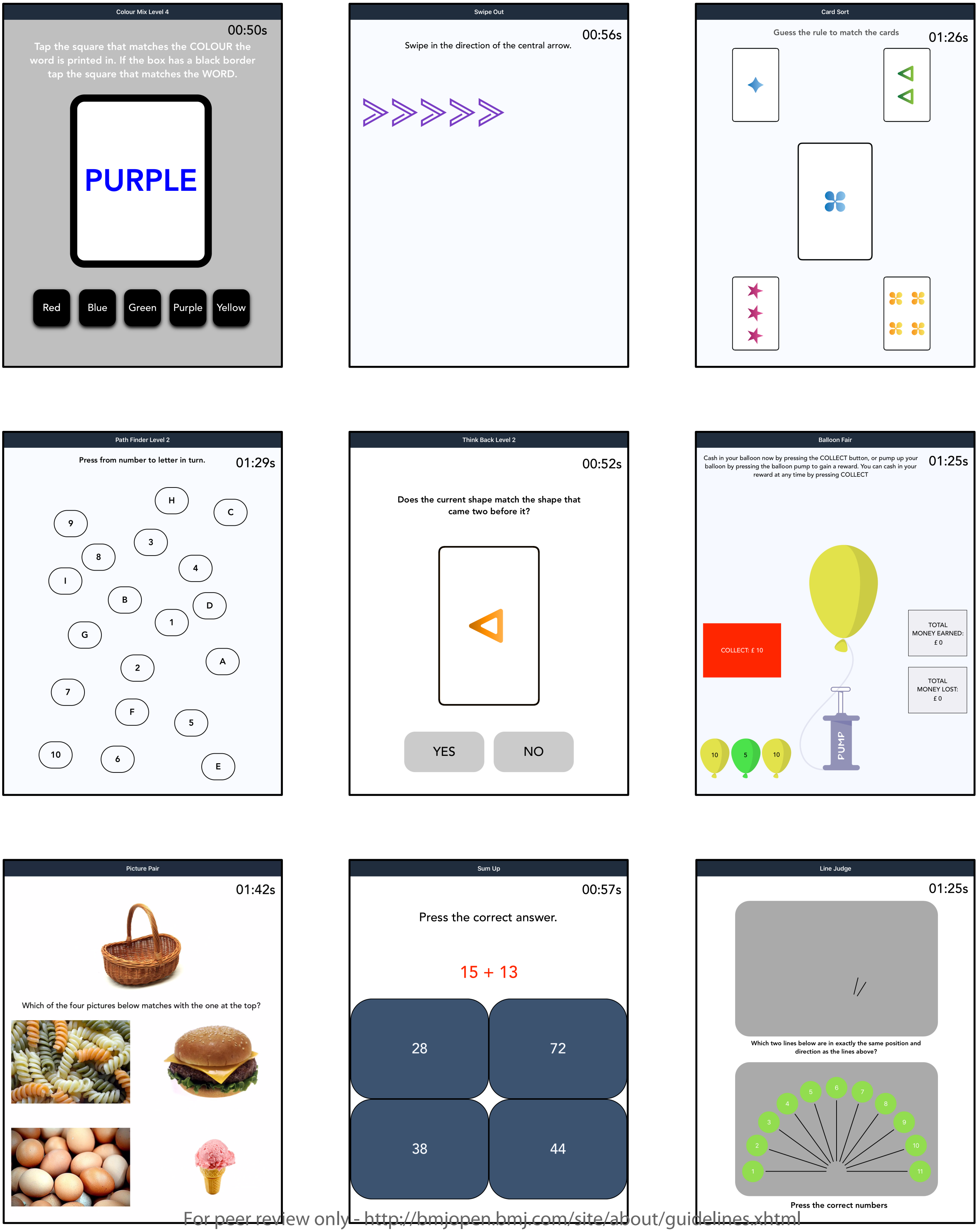

For peer review only - http://bmjopen.bmj.com/site/about/guidelines.xhtml

BMJ Open

Study protocol: computerized cognitive testing in a cohort of people with frontotemporal dementia

Journal:	*BMJ Open*
Manuscript ID	bmjopen-2021-055211.R2
Article Type:	Protocol
Date Submitted by the Author:	13-May-2022
Complete List of Authors:	Moore, Katrina; UCL Queen Square Institute of Neurology, Department of Neurodegenerative Disease<br>Convery, Rhian S.; UCL Queen Square Institute of Neurology, Department of Neurodegenerative Disease<br>Rohrer, Jonathan; UCL Queen Square Institute of Neurology, Department of Neurodegenerative Disease
**Primary Subject Heading**:	Neurology
Secondary Subject Heading:	Genetics and genomics
Keywords:	Dementia < NEUROLOGY, Adult neurology < NEUROLOGY, Delirium & cognitive disorders < PSYCHIATRY

SCHOLARONE™
Manuscripts

STUDY PROTOCOL

Main title

Study protocol: computerized cognitive testing in a cohort of people with frontotemporal dementia

Authors

Katrina M Moore PhD[1], Rhian S. Convery MSc[1], Jonathan D Rohrer FRCP PhD[1]

Affiliations

[1]Dementia Research Centre, Department of Neurodegenerative Disease, UCL Queen Square Institute of Neurology, London, UK.

Corresponding author

Professor Jonathan Rohrer, Dementia Research Centre, Department of Neurodegenerative Disease, UCL Institute of Neurology, Queen Square, London, WC1N 3BG, j.rohrer@ucl.ac.uk

For peer review only

Abstract

Introduction: The term frontotemporal dementia (FTD) refers to a heterogeneous group of neurodegenerative disorders affecting the frontal and temporal lobes. Cognitively, impairment of executive function and social cognition predominate across the FTD spectrum although other domains can be affected. Traditionally, cognition is tested through standard 'pen and paper' tasks in FTD. However, recent attempts have been made across other neurodegenerative disorders such as Alzheimer's disease to develop computerized batteries that allow more accurate and sensitive detection of cognitive impairment.

Methods and analysis: This paper describes the development of a novel battery of tests for a tablet computer, particularly focused on FTD. It consists of twelve different tasks which aim to tap into information processing speed, various aspects of executive function, social cognition, semantic knowledge, calculation and visuospatial skills. Future studies will focus on validating the battery in a healthy control cohort, comparing it against a standard 'pen and paper' psychometric battery, and finally testing it within an FTD cohort, including those with genetic forms of FTD where we will be able to assess its ability to detect very early cognitive deficits prior to the onset of symptoms.

Ethics and dissemination: Normative data will be produced in the initial validation study and will be made available online.

Keywords: frontotemporal dementia, progranulin, tau, C9orf72, cognition

For peer review only

STRENGTHS AND LIMITATIONS

- This is one of the first computerized cognitive batteries to focus specifically on FTD.
- Tests are short and can be performed at home, reducing participant burden within studies.
- The study will require validation including comparison with a gold standard 'pen and paper' psychometric battery before it can be used more widely.

For peer review only

INTRODUCTION

Frontotemporal dementia (FTD) is a clinically, pathologically and genetically diverse neurodegenerative disease.[1] It is probably the commonest form of dementia in those under 60 years of age and is associated with two main clinical presentations: an impairment in personality and social conduct, known as behavioural variant FTD (bvFTD), and deficits in speech and language, called primary progressive aphasia (PPA). Around a third of FTD is familial,[2] with the majority of autosomal dominant FTD linked to mutations in three key genes: chromosome 9 open reading frame 72 (*C9orf72*), progranulin (*GRN*) and microtubule-associated protein tau (*MAPT*).

The cognitive deficits found in FTD are commonly in the executive function and social cognition domains for bvFTD,[3-5] and the language and semantic knowledge domains for those with PPA, [5-8] although there is often overlap between these disorders. Classically, episodic memory and more posterior cortical functions are spared, particularly in the early stages of the disease.[9] The trajectory of cognitive impairment has now been studied across a number of cohorts, with the earliest change being studied mostly within genetic cohorts where at-risk individuals who are many years from expected symptom onset can be investigated. Deficits across several cognitive tasks have been shown around five to eight years prior to symptom onset particularly in tests of executive function and social cognition, although the pattern of deficits can differ according to which mutation is carried.[10-16] However, prior studies have been performed mainly using traditional 'pen and paper' tasks which may well be limited in their ability to detect subtle changes. Using magnetic resonance imaging, neuroanatomical changes can be seen in presymptomatic genetic FTD studies much earlier, potentially over 20 years prior to expected symptom onset,[12] and so there is a need to bridge the gap between the sensitivity of neuroimaging techniques and current cognitive assessments.

For peer review only

Treatment trials in FTD are currently underway, and although many of the planned studies are focused on symptomatic FTD, it is likely that future studies will also include people in the presymptomatic phase of the disease. One of the greatest challenges facing trials of this nature is having robust cognitive outcome measures. The Food and Drug Administration have recognized that current assessments are not optimal, highlighting the need for improved cognitive markers as outcome measures for clinical trials,[17] and have encouraged the development of novel approaches to evaluate subtle deficits that may emerge in the presymptomatic stages of dementia.[18] Here we describe the design and protocol of Ignite, a novel computerized cognitive assessment tool designed for FTD. We outline the key objectives for developing a computerized battery and provide an overview of the cognitive domains tested within the battery.

METHODS AND ANALYSIS

Patient and Public Involvement

It is standard for traditional cognitive assessments to be performed by trained psychologists in hospital or research settings, which can be both time consuming and costly. On discussion with research participants they describe frequently experiencing high levels of anxiety in relation to attending research visits, particularly in relation to neuropsychological asssessment. In presymptomatic FTD studies this can be most prominent in those who have yet to find out their carrier status. In addition, the number of in person contact days required for a clinical trial can be burdensome and can prevent an individual's participation. With these considerations in mind, we wanted to design a computerized battery that could be performed by individuals themselves, with the aim of reducing participant anxiety, thus ensuring that their best performance is captured. We therefore initially discussed the development of this battery with a small group of people at-risk of familial FTD to ensure we could maximize the user-friendliness of the battery and reduce the participant burden.

For peer review only

Objectives for developing a computerized cognitive battery

Apart from improving the participant experience, we had a number of other objectives for developing the battery:

Comprehensiveness

We wanted to develop a set of assessments that could capture an individual's performance across a number of cognitive domains in a comprehensive manner for FTD. A major drawback of current cognitive assessments is the duration of testing required to get an accurate picture of individual performance, often taking a number of hours. The new battery was designed to be completed in around 30 minutes or less.

Increased sensitivity

Ignite consists mainly of tests which are modifications of existing cognitive tasks. However, each task was modified to shorten the time allowed for completion, with the measurement of reaction times aiming to increase the sensitivity to detect deficits.

Self-assessment

A key aim in the development of Ignite was its ability to be performed by individuals on their own, ideally while at home. All assessments were designed to be as intuitive as possible, appearing more like short games than standard cognitive tests, including the use of simple on-screen buttons or swipes to record answers. A challenge in designing tasks with self-assessment in mind is ensuring that participants are motivated to complete the tasks on their own and that they understand what is being asked of them. To assist participants in understanding and remembering task instructions, they are provided with brief written instructions prior to beginning each individual assessment task. Accompanying the written

For peer review only

instructions are short videos to ensure participants can clearly visualize and understand what is required of them. In addition, once a task has begun, a prompt of the task instruction is displayed at the top of the screen for the duration of the task. In this way, participants can refresh their memories of the task instructions at any time without having to exit the task. To keep participants motivated and engaged each task was designed to range from 30-180 seconds in duration, with most tasks programmed to time out after 60 seconds.

Protocol

Ignite consists of twelve separate assessments that measure a spectrum of cognitive functions (Table 1, Figure 1). Each test was included based on knowledge of the deficits seen in FTD, and in particular those domains most likely to be affected presymptomatically. The tests are self-administered in a predetermined order, in an environment familiar to the participant, such as their home. At present, the app is available on the iPad via the App Store, although future iterations will aim to make versions available for other tablets and operating systems.

Executive function

The term executive function describes a number of different aspects of cognition including reasoning, problem solving and planning [19] commonly related to the workings of the frontal lobe. It consists of a number of different subdomains, which include inhibitory control, cognitive flexibility and working memory.[20] We aimed to design tasks tapping into each of these subdomains as well as decision making and cognitive timing as previous studies of FTD have shown deficits within all of them,[21-23] and in particular impairment can be seen presymptomatically across each of the major genetic groups.[12,24]

Inhibitory control

For peer review only

This subdomain is described as the ability to control one's attention, behaviour and thoughts to override a strong internal predisposition or external lure.[20] It is abnormal in FTD and relates to the key symptom of disinhibition that forms part of the diagnostic criteria for behavioural variant FTD.[25] We included two tasks of inhibitory control.

Colour Mix

In our task, we adapted the classical Stroop task including five different colours: red, blue, green, purple and yellow. The initial two levels of the task test speed of processing. In Level 1, participants are presented with a coloured circle and all five colour names written below. They are asked to match the colour of the circle with the correct colour name from 5. They have 30 seconds to complete as many as possible (with a maximum of 50 possible trials). In Level 2, participants are presented with the name of a colour and (as with Level 1) the five colour names written below. They are asked to match the target colour word with the matching word below. They have 30 seconds to complete as many as possible (with a maximum of 50 possible trials). The next two levels measure inhibitory control. In Level 3, participants are presented with a colour word written in a different colour e.g. BLUE written in red ink. They are asked to match the colour of the ink (rather than the name) to one of the five colour names written below. They have 60 seconds to complete as many as possible (with a maximum of 50 possible trials). Lastly, in Level 4, participants are presented as in Level 3 with a colour word written in a different colour, and have to complete the task as in Level 3, unless a black border appears around the word (as it does on some trials), when they have to match the written word rather than the ink colour (e.g. match blue if the word is BLUE written in red ink) to one of the five colour names written below. They have 60 seconds to complete as many as possible (with a maximum of 50 possible trials).

Swipe Out

For peer review only

Our task was a modification of the classical Flanker task, with an arrowhead (facing up, down, left or right) with four flanking arrowheads surrounding the target arrowhead. Participants are asked to swipe in the direction of the central arrowhead. In some trials the flanking arrowheads are facing in the same direction, and in some trials they are facing in the opposite direction. Participants have 60 seconds to complete as many trials as possible (with a maximum of 40 possible trials).

Cognitive flexibility

This subdomain describes the ability to change perspectives from an established pattern, and mentally adjust or 'set shift' our perspective to new task demands. Tasks that tap into cognitive flexibility include the Wisconsin Card Sorting Task (WCST) [26] and the Trail Making Test (TMT) [27]. Deficits in both of these tasks have been previously described in FTD [10,13,28] including presymptomatically in those with genetic forms of FTD.[29]

Card Sort

Our task adapts a classical card sorting task, asking participants to sort cards by dragging them from the centre of the screen into card deposits located in the four corners of the screen. Participants are required to sort cards by a rule: either shape, number or colour. The primary aim of the task is for the participant to understand the correct sorting criteria on the basis of feedback, either correct or incorrect, and to switch flexibly between sorting rules whenever feedback indicates that the rule has changed. After a set number of correct card sorts are performed the rule changes, and negative feedback is given if participants continue to sort by the previous rule. The successful performance of the task is measured in participants' ability to flexibly respond to feedback given in shifting to the new rule.[26] The number of perseverative errors, identified by participants failing to adapt to a new rule, is thought to be indicative of poor cognitive

For peer review only

flexibility.[30] In our task, participants have a total of 90 seconds in order to sort as many as cards as possible.

Path Finder

This task adapts the Trail Making Test (TMT), asking participants to tap stimuli in sequential order (rather than draw an unbroken line between letters and/or numbers as in the original pen and paper task). Correct sequencing is indicated by the outline of each letter or number changing colour to green. The letter or number changes to red if selected incorrectly. In Level 1 participants are asked to select numbers in the correct order (similar to TMT part A), with a maximum of 60 seconds to complete the task. In Level 2 participants are asked to alternate between numbers and letters in the correct order (similar to TMT part B), with a maximum of 90 seconds to complete the task.

Working memory

This subdomain of executive function describes the ability to hold information in mind and mentally work with it.[31] A classic paradigm for assessing working memory is the N-back task, which requires participants to monitor a series of stimuli and respond whenever the stimuli presented matches the properties of the trial presented 'N' before it.[32] The task requires the monitoring, updating and manipulation of information displayed to perform the task effectively.

Think Back

There are two levels to our N-Back task. Level 1 is a 1-back task and Level 2 is a 2-back task. In Level 1 participants must indicate whether a shape matches the one preceding it. In Level 2 participants must indicate whether a shape matches the shape shown two before it, intended to place greater demand on

For peer review only

working memory. An answer is submitted by pressing a 'yes' or 'no' button. In each level, participants have 60 seconds to complete as many trials as possible (up to a maximum of 72 trials).

Decision making

For many people with FTD their ability to make decisions is compromised.[33] Several paradigms are employed in assessing decision making behaviour, often in the form of gambling or risk-taking tasks, such as the Iowa Gambling Task,[34] Cambridge Gambling Task,[35] Game of Dice Task [36] and the Balloon Analogue Risk Task,[37] with people with FTD shown to be impaired on these tests.[38]

Balloon Fair

In our task we adapted the Balloon Analogue Risk Task [37] to assess decision making behaviour. Participants are presented with a simulated balloon and balloon pump. Also presented is a button labelled 'Collect £' and a permanent money counter labelled 'Total Earned', alongside a counter displaying money that has been lost. With each press on the pump, the balloon is inflated, and money is accumulated in a temporary bank. Balloons pop after a predetermined 'popping' point, at which point the money accrued in the temporary bank is lost. Participants have the option to cash in the money accumulated in the temporary bank at any point by clicking the 'Collect £' button. After each balloon has popped (money lost) or the money has been collected a new balloon appears. Balloons are differently coloured (green, yellow and blue) with each colour associated with a unique sum (£5, £10, £50 respectively), and a unique popping point (quicker for higher sums). Participants are unaware of the probability of a balloon popping. The aim of the task is to earn as much money as possible. Participants have 90 seconds to complete the task.

Cognitive timing

For peer review only

The concept of timing is integral to performing many mental processes.[39] Although not well-studied in FTD, one previous study has shown impairment in both externally-paced and self-paced finger tapping.[23]

Time Tap

We adapted the task described in Henley et al., 2014 [23] to test cognitive timing. A pulsating circle is presented in the middle of the top of the screen and a tone sounds in time to the pulse (at 1500ms intervals). Participants are asked to use the index finger of their dominant hand to tap in time with the pulsating circle on a second circle presented below. After 30 seconds the pulsation and tone cease, and participants are instructed to maintain the same tempo for a further 30 seconds.

Social Cognition

Social cognition is the ability to perceive, interpret and generate a response to the intentions, behaviours, and feelings of others, and includes a number of subdomains such as emotion recognition and theory of mind. Social cognition impairment occurs early and almost universally in all people with FTD [40,41] as well as those with other forms of FTD, such as PPA.[42,43] These deficits include poor recognition of simple emotions in faces,[44,45] as well as impairment of complex emotion processing, and other tasks of theory of mind [45,46] Early deficits have been seen in genetic FTD including those who are presymptomatic.[13]

Face Match

In our task of simple emotion processing participants are presented with a target basic emotion word (sadness, happiness, anger, fear, disgust, surprise) at the top of the screen alongside nine images of faces in the corners of the screen.[47] Participants are asked to tap all of the faces displaying the target emotion as quickly as possible. There are five correct answers out of each nine presented faces, and participants

For peer review only

must press all five within 10 seconds, or the task moves on to the next set of nine faces. The participants have to complete as many items as possible within 60 seconds (up to a maximum of 6 sets of nine faces).

Mind Reading

Our task of complex emotion processing adapts the Reading the Mind in the Eyes test so that participants are presented with a target emotion word in the centre of the screen alongside four images of eyes in the corners of the screen, one of which matches the emotion. Participants are asked to select the image which best matches the target emotion word, completing as many as possible within 90 seconds (up to a maximum of 20 words).

Semantic knowledge

Impairment of semantic knowledge is characteristic of the semantic variant of primary progressive aphasia but is seen in other forms of FTD, and has been shown to occur presymptomatically, particularly in those with *MAPT* mutations.[48,49]

Picture Pair

Our task uses stimuli from the modified version of the Camel and Cactus task that we have previously described as detecting early semantic deficits in genetic FTD.[50] The participant has to match a target picture with a semantically-linked matching picture from a choice of four e.g. matching Camel with Cactus rather than a tree, sunflower or rose. The participants have to answer as many questions as possible in 120 seconds (with a maximum of 25 questions).

Arithmetic

For peer review only

Calculation abilities have been shown to be highly dependent on left parietal lobe function.[51,52] Although initial studies focused on anterior involvement of the frontal and temporal lobes in FTD, more recent studies have shown that some forms of FTD can have more posterior cortical atrophy, particularly those with *GRN* mutations and *C9orf72* expansions,[53] including presymptomatically.[12] Dyscalculia has been reported in both of these groups.[54,55] Although deficits are generally not limited to one form of mental arithmetic, one study has shown deficits specifically in multiplication in ALS.[56]

Sum Up

Our task includes additions, subtractions, multiplications and divisions, with participants presented with the sum and four possible answers, and a time limit of 10 seconds for completion of each sum. The participants have to answer as many questions as possible within 60 seconds (with a maximum of 48 sums, 12 of each type).

Visuospatial skills

Visuospatial processing is classically associated with right parietal lobe function.[57] As mentioned above, more posterior cortical atrophy, including involvement of the right parietal lobe has been seen in genetic FTD. Given this finding, it is unsurprising that several studies have highlighted impairments of visuospatial skills in both *GRN* mutation carriers [58,59] and *C9orf72* expansion carriers.[60]

Line Judge

Judgement of line orientation is a standard measure of visuospatial skills,[61,62] testing an individual's ability to match the angle and orientation of lines in space, with deficits seen in forms of dementia such as Alzheimer's diseases and Dementia with Lewy Bodies which are usually associated with parietal atrophy.[63,64]

For peer review only

In our task, participants are presented with pairs of angled lines and are asked to match the target pair of lines to a set of 11 lines arranged in a semicircle and numbered 1 to 11. Participants press the correct numbers that correspond to the line pair. The participants have to answer as many questions as possible within a 90 second timeframe (up to a maximum of 15 pairs).

Ethics and dissemination

Ignite is a novel computerized battery of assessments for the detection of cognitive changes in FTD. It includes tasks of information processing speed, executive function, social cognition, semantic knowledge, arithmetic and visuospatial skills. It potentially may be sensitive enough to detect early (and presymptomatic) deficits within an at-risk genetic cohort. Ignite will be initially validated in a healthy control cohort and compared against a standard set of 'pen and paper' psychometric tasks, including assessment of test-retest reliability. Normative data will be produced in this initial validation study and will be made available online. The battery will then be tested within an FTD cohort such as GENFI (www.genfi.org; [12]) where it would be possible to assess both presymptomatic and symptomatic people with FTD. In the future we would also hope to be able to compare Ignite with other emerging computerized batteries aimed specifically at FTD, such as those being developed as part of the ARTFL-LEFFTDS Longitudinal Frontotemporal Lobar Degeneration (ALLFTD: www.allftd.org) study. The final goal of the Ignite study will be to develop a set of validated tasks which are able to track disease progression in FTD, specifically in the presymptomatic and very early symptomatic phase of the disease, and therefore have the potential to be employed in clinical trials as a cognitive outcome measure.

For peer review only

Abbreviations

FTD, frontotemporal dementia; PPA, primary progressive aphasia; C9orf72, chromosome 9 open reading frame 72; GRN, progranulin; MAPT, microtubule-associated protein tau; bvFTD, behavioural variant frontotemporal dementia, TMT, Trail Making Test; WCST, Wisconsin Card Sorting Task.

For peer review only

Declarations

Ethics approval and consent to participate

The study has been approved by the UCL ethics committee. Written informed consent is being obtained for those taking part in the study.

Consent to publish

Not applicable

Availability of data and materials

The Ignite app is free to download and use from the Appstore.

Competing interests

KMM is now an employee of Ionis Pharmaceuticals. JDR has served on medical advisory boards or provided consultancy for Alector, Arkuda Therapeutics, Wave Life Sciences, Prevail Therapeutics, UCB, AC Immune, Astex Pharmaceuticals, Biogen, Takeda and Eisai.

Funding

The pilot version of the app was developed through an Alzheimer's Society PhD studentship (for KMM) awarded to JDR (AS-PhD-2015-005), with further development funded by a Frontotemporal Dementia Research Studentship in Memory of David Blechner awarded through The National Brain Appeal (RCN 290173) to RSC. JDR has received funding from an MRC Clinician Scientist Fellowship (MR/M008525/1), an NIHR Rare Disease Translational Research Collaboration (BRC149/NS/MH) and the Brain Research UK Miriam Marks Senior Fellowship; his work is also supported by the MRC UK GENFI grant (MR/M023664/1), the Bluefield Project and the JPND GENFI-PROX grant (2019-02248). None of the funding bodies had any direct input into the app development.

Authors' contributions

KMM, RSC and JDR wrote the manuscript. All authors have read and approved the manuscript.

Acknowledgements

We thank the research participants at the Dementia Research Centre for their contribution to the development of Ignite.

For peer review only

References

1. Warren, J. D., Rohrer, J. D., & Rossor, M. N. (2013). Clinical review. Frontotemporal dementia. BMJ (Clinical research ed.), 347, f4827.

2. Rohrer, J. D., Guerreiro, R., Vandrovcova, J., Uphill, J., Reiman, D., Beck, J., Isaacs, A. M., Authier, A., Ferrari, R., Fox, N. C., Mackenzie, I. R., Warren, J. D., de Silva, R., Holton, J., Revesz, T., Hardy, J., Mead, S., & Rossor, M. N. (2009). The heritability and genetics of frontotemporal lobar degeneration. Neurology, 73(18), 1451–1456.

3. Grossman M. (2002). Frontotemporal dementia: a review. Journal of the International Neuropsychological Society : JINS, 8(4), 566–583.

4. Harciarek, M., & Jodzio, K. (2005). Neuropsychological differences between frontotemporal dementia and Alzheimer's disease: a review. Neuropsychology review, 15(3), 131–145.

5. Harciarek, M., & Cosentino, S. (2013). Language, executive function and social cognition in the diagnosis of frontotemporal dementia syndromes. International review of psychiatry (Abingdon, England), 25(2), 178–196.

6. Warren, J. D., Rohrer, J. D., & Rossor, M. N. (2013). Clinical review. Frontotemporal dementia. BMJ (Clinical research ed.), 347, f4827.

7. Woollacott, I. O., & Rohrer, J. D. (2016). The clinical spectrum of sporadic and familial forms of frontotemporal dementia. Journal of neurochemistry, 138 Suppl 1, 6–31.

8. Deleon, J., & Miller, B. L. (2018). Frontotemporal dementia. Handbook of clinical neurology, 148, 409–430.

9. Gorno-Tempini, M. L., Hillis, A. E., Weintraub, S., Kertesz, A., Mendez, M., Cappa, S. F., Ogar, J. M., Rohrer, J. D., Black, S., Boeve, B. F., Manes, F., Dronkers, N. F., Vandenberghe, R., Rascovsky, K., Patterson, K., Miller, B. L., Knopman, D. S., Hodges, J. R., Mesulam, M. M., & Grossman, M.

For peer review only

(2011). Classification of primary progressive aphasia and its variants. Neurology, 76(11), 1006–1014.

10. Geschwind, D. H., Robidoux, J., Alarcón, M., Miller, B. L., Wilhelmsen, K. C., Cummings, J. L., & Nasreddine, Z. S. (2001). Dementia and neurodevelopmental predisposition: cognitive dysfunction in presymptomatic subjects precedes dementia by decades in frontotemporal dementia. Annals of neurology, 50(6), 741–746.

11. Hallam, B. J., Jacova, C., Hsiung, G. Y., Wittenberg, D., Sengdy, P., Bouchard-Kerr, P., Slack, P., Rademakers, R., Baker, M., Chow, T. W., Levine, B., Feldman, H. H., & Mackenzie, I. R. (2014). Early neuropsychological characteristics of progranulin mutation carriers. Journal of the International Neuropsychological Society : JINS, 20(7), 694–703.

12. Rohrer, J. D., Nicholas, J. M., Cash, D. M., van Swieten, J., Dopper, E., Jiskoot, L., van Minkelen, R., Rombouts, S. A., Cardoso, M. J., Clegg, S., Espak, M., Mead, S., Thomas, D. L., De Vita, E., Masellis, M., Black, S. E., Freedman, M., Keren, R., MacIntosh, B. J., Rogaeva, E., … Binetti, G. (2015). Presymptomatic cognitive and neuroanatomical changes in genetic frontotemporal dementia in the Genetic Frontotemporal dementia Initiative (GENFI) study: a cross-sectional analysis. The Lancet. Neurology, 14(3), 253–262.

13. Jiskoot, L. C., Dopper, E. G., Heijer, T. d., Timman, R., van Minkelen, R., van Swieten, J. C., & Papma, J. M. (2016). Presymptomatic cognitive decline in familial frontotemporal dementia: A longitudinal study. Neurology, 87(4), 384–391.

14. Papma, J. M., Jiskoot, L. C., Panman, J. L., Dopper, E. G., den Heijer, T., Donker Kaat, L., Pijnenburg, Y., Meeter, L. H., van Minkelen, R., Rombouts, S., & van Swieten, J. C. (2017). Cognition and gray and white matter characteristics of presymptomatic C9orf72 repeat expansion. Neurology, 89(12), 1256–1264.

For peer review only

15. Suhonen, N. M., Haanpää, R. M., Korhonen, V., Jokelainen, J., Pitkäniemi, A., Heikkinen, A. L., Krüger, J., Hartikainen, P., Helisalmi, S., Hiltunen, M., Hänninen, T., & Remes, A. M. (2017). Neuropsychological Profile in the C9ORF72 Associated Behavioral Variant Frontotemporal Dementia. Journal of Alzheimer's disease : JAD, 58(2), 479–489.

16. Jiskoot, L. C., Panman, J. L., van Asseldonk, L., Franzen, S., Meeter, L., Donker Kaat, L., van der Ende, E. L., Dopper, E., Timman, R., van Minkelen, R., van Swieten, J. C., van den Berg, E., & Papma, J. M. (2018). Longitudinal cognitive biomarkers predicting symptom onset in presymptomatic frontotemporal dementia. Journal of neurology, 265(6), 1381–1392.

17. Sabbagh, M. N., Hendrix, S., & Harrison, J. E. (2019). FDA position statement "Early Alzheimer's disease: Developing drugs for treatment, Guidance for Industry". Alzheimer's & dementia (New York, N. Y.), 5, 13–19.

18. FDA (2018). Early Alzheimer's Disease: Developing Drugs for Treatment Guidance for Industry DRAFT GUIDANCE. Retrieved from https://www.fda.gov/Drugs/GuidanceComplianceRegulatoryInformation/Guidances/default.htm

19. Collins, A., & Koechlin, E. (2012). Reasoning, learning, and creativity: frontal lobe function and human decision-making. PLoS biology, 10(3), e1001293.

20. Diamond A. (2013). Executive functions. Annual review of psychology, 64, 135–168.

21. Strenziok, M., Pulaski, S., Krueger, F., Zamboni, G., Clawson, D., & Grafman, J. (2011). Regional brain atrophy and impaired decision making on the balloon analog risk task in behavioral variant frontotemporal dementia. Cognitive and behavioral neurology : official journal of the Society for Behavioral and Cognitive Neurology, 24(2), 59–67.

22. Manes, F., Torralva, T., Ibáñez, A., Roca, M., Bekinschtein, T., & Gleichgerrcht, E. (2011). Decision-making in frontotemporal dementia: clinical, theoretical and legal implications. Dementia and geriatric cognitive disorders, 32(1), 11–17.

For peer review only

23. Henley, S. M., Downey, L. E., Nicholas, J. M., Kinnunen, K. M., Golden, H. L., Buckley, A., Mahoney, C. J., & Crutch, S. J. (2014). Degradation of cognitive timing mechanisms in behavioural variant frontotemporal dementia. Neuropsychologia, 65, 88–101.

24. Staffaroni, A. M., Bajorek, L., Casaletto, K. B., Cobigo, Y., Goh, S. M., Wolf, A., Heuer, H. W., Elahi, F. M., Ljubenkov, P. A., Dever, R., Kornak, J., Appleby, B., Bove, J., Bordelon, Y., Brannelly, P., Brushaber, D., Caso, C., Coppola, G., Dheel, C., Dickerson, B. C., … ARTFL/LEFFTDS consortium (2020). Assessment of executive function declines in presymptomatic and mildly symptomatic familial frontotemporal dementia: NIH-EXAMINER as a potential clinical trial endpoint. Alzheimer's & dementia : the journal of the Alzheimer's Association, 16(1), 11–21.

25. Rascovsky, K., Hodges, J. R., Knopman, D., Mendez, M. F., Kramer, J. H., Neuhaus, J., van Swieten, J. C., Seelaar, H., Dopper, E. G., Onyike, C. U., Hillis, A. E., Josephs, K. A., Boeve, B. F., Kertesz, A., Seeley, W. W., Rankin, K. P., Johnson, J. K., Gorno-Tempini, M. L., Rosen, H., Prioleau-Latham, C. E., … Miller, B. L. (2011). Sensitivity of revised diagnostic criteria for the behavioural variant of frontotemporal dementia. Brain : a journal of neurology, 134(Pt 9), 2456–2477.

26. Stuss, D. T., Levine, B., Alexander, M. P., Hong, J., Palumbo, C., Hamer, L., Murphy, K. J., & Izukawa, D. (2000). Wisconsin Card Sorting Test performance in patients with focal frontal and posterior brain damage: effects of lesion location and test structure on separable cognitive processes. Neuropsychologia, 38(4), 388–402.

27. Delis, D. ., Kaplan, E., & J.H, K. (2001). The Delis-Kaplan Executive Function System: Examiner's Manual. San Andonio: The Psychological Corporation.

28. Rohrer, J. D., Warren, J. D., Omar, R., Mead, S., Beck, J., Revesz, T., Holton, J., Stevens, J. M., Al-Sarraj, S., Pickering-Brown, S. M., Hardy, J., Fox, N. C., Collinge, J., Warrington, E. K., & Rossor, M. N. (2008). Parietal lobe deficits in frontotemporal lobar degeneration caused by a mutation in the progranulin gene. Archives of neurology, 65(4), 506–513.

For peer review only

29. Barandiaran, M., Estanga, A., Moreno, F., Indakoetxea, B., Alzualde, A., Balluerka, N., Martí Massó, J. F., & López de Munain, A. (2012). Neuropsychological features of asymptomatic c.709-1G>A progranulin mutation carriers. Journal of the International Neuropsychological Society : JINS, 18(6), 1086–1090.

30. Lange, F., Brückner, C., Knebel, A., Seer, C., & Kopp, B. (2018). Executive dysfunction in Parkinson's disease: A meta-analysis on the Wisconsin Card Sorting Test literature. Neuroscience and biobehavioral reviews, 93, 38–56.

31. Baddeley A.D, & Hitch G.J. (1994). Developments in the Concept of Working Memory. Neuropsychology, 8, 485–493.

32. Owen, A. M., McMillan, K. M., Laird, A. R., & Bullmore, E. (2005). N-back working memory paradigm: a meta-analysis of normative functional neuroimaging studies. Human brain mapping, 25(1), 46–59.

33. Gleichgerrcht, E., Torralva, T., Roca, M., Szenkman, D., Ibanez, A., Richly, P., Pose, M., & Manes, F. (2012). Decision making cognition in primary progressive aphasia. Behavioural neurology, 25(1), 45–52.

34. Bechara, A., & Van Der Linden, M. (2005). Decision-making and impulse control after frontal lobe injuries. Current opinion in neurology, 18(6), 734–739.

35. Rogers, R. D., Owen, A. M., Middleton, H. C., Williams, E. J., Pickard, J. D., Sahakian, B. J., & Robbins, T. W. (1999). Choosing between small, likely rewards and large, unlikely rewards activates inferior and orbital prefrontal cortex. The Journal of neuroscience : the official journal of the Society for Neuroscience, 19(20), 9029–9038.

36. Brand, M., Fujiwara, E., Borsutzky, S., Kalbe, E., Kessler, J., & Markowitsch, H. J. (2005). Decision-making deficits of korsakoff patients in a new gambling task with explicit rules: associations with executive functions. Neuropsychology, 19(3), 267–277.

For peer review only

37. Lejuez, C. W., Read, J. P., Kahler, C. W., Richards, J. B., Ramsey, S. E., Stuart, G. L., Strong, D. R., & Brown, R. A. (2002). Evaluation of a behavioral measure of risk taking: the Balloon Analogue Risk Task (BART). Journal of experimental psychology. Applied, 8(2), 75–84.

38. Rahman, S., Sahakian, B. J., Hodges, J. R., Rogers, R. D., & Robbins, T. W. (1999). Specific cognitive deficits in mild frontal variant frontotemporal dementia. Brain : a journal of neurology, 122 (Pt 8), 1469–1493.

39. Allman, M. J., & Meck, W. H. (2012). Pathophysiological distortions in time perception and timed performance. Brain : a journal of neurology, 135(Pt 3), 656–677.

40. Snowden, J. S., Gibbons, Z. C., Blackshaw, A., Doubleday, E., Thompson, J., Craufurd, D., Foster, J., Happé, F., & Neary, D. (2003). Social cognition in frontotemporal dementia and Huntington's disease. Neuropsychologia, 41(6), 688–701.

41. Russell, L. L., Greaves, C. V., Bocchetta, M., Nicholas, J., Convery, R. S., Moore, K., Cash, D. M., van Swieten, J., Jiskoot, L., Moreno, F., Sanchez-Valle, R., Borroni, B., Laforce, R., Jr, Masellis, M., Tartaglia, M. C., Graff, C., Rotondo, E., Galimberti, D., Rowe, J. B., Finger, E., … Genetic FTD Initiative, GENFI (2020). Social cognition impairment in genetic frontotemporal dementia within the GENFI cohort. Cortex; a journal devoted to the study of the nervous system and behavior, 133, 384–398.

42. Couto, B., Manes, F., Montañés, P., Matallana, D., Reyes, P., Velasquez, M., Yoris, A., Baez, S., & Ibáñez, A. (2013). Structural neuroimaging of social cognition in progressive non-fluent aphasia and behavioral variant of frontotemporal dementia. Frontiers in human neuroscience, 7, 467.

43. Fittipaldi, S., Ibanez, A., Baez, S., Manes, F., Sedeno, L., & Garcia, A. M. (2019). More than words: Social cognition across variants of primary progressive aphasia. Neuroscience and biobehavioral reviews, 100, 263–284.

For peer review only

44. Kumfor, F., & Piguet, O. (2012). Disturbance of emotion processing in frontotemporal dementia: a synthesis of cognitive and neuroimaging findings. Neuropsychology review, 22(3), 280–297.

45. Lough, S., Kipps, C. M., Treise, C., Watson, P., Blair, J. R., & Hodges, J. R. (2006). Social reasoning, emotion and empathy in frontotemporal dementia. Neuropsychologia, 44(6), 950–958.

46. Gregory, C., Lough, S., Stone, V., Erzinclioglu, S., Martin, L., Baron-Cohen, S., & Hodges, J. R. (2002). Theory of mind in patients with frontal variant frontotemporal dementia and Alzheimer's disease: theoretical and practical implications. Brain : a journal of neurology, 125(Pt 4), 752–764.

47. Tottenham, N., Tanaka, J. W., Leon, A. C., McCarry, T., Nurse, M., Hare, T. A., Marcus, D. J., Westerlund, A., Casey, B. J., & Nelson, C. (2009). The NimStim set of facial expressions: judgments from untrained research participants. Psychiatry research, 168(3), 242–249.

48. Snowden, J. S., Adams, J., Harris, J., Thompson, J. C., Rollinson, S., Richardson, A., Jones, M., Neary, D., Mann, D. M., & Pickering-Brown, S. (2015). Distinct clinical and pathological phenotypes in frontotemporal dementia associated with MAPT, PGRN and C9orf72 mutations. Amyotrophic lateral sclerosis & frontotemporal degeneration, 16(7-8), 497–505.

49. Hardy, C. J., Buckley, A. H., Downey, L. E., Lehmann, M., Zimmerer, V. C., Varley, R. A., Crutch, S. J., Rohrer, J. D., Warrington, E. K., & Warren, J. D. (2016). The Language Profile of Behavioral Variant Frontotemporal Dementia. Journal of Alzheimer's disease : JAD, 50(2), 359–371.

50. Moore, K., Convery, R., Bocchetta, M., Neason, M., Cash, D. M., Greaves, C., Russell, L. L., Clarke, M., Peakman, G., van Swieten, J., Jiskoot, L., Moreno, F., Barandiaran, M., Sanchez-Valle, R., Borroni, B., Laforce, R., Jr, Doré, M. C., Masellis, M., Tartaglia, M. C., Graff, C., … Genetic FTD Initiative, GENFI* (2020). A modified Camel and Cactus Test detects presymptomatic semantic impairment in genetic frontotemporal dementia within the GENFI cohort. Applied neuropsychology. Adult, 1–8.

For peer review only

51. Takayama, Y., Sugishita, M., Akiguchi, I., & Kimura, J. (1994). Isolated acalculia due to left parietal lesion. Archives of neurology, 51(3), 286–291.

52. Dehaene, S., Piazza, M., Pinel, P., & Cohen, L. (2003). Three parietal circuits for number processing. Cognitive neuropsychology, 20(3), 487–506.

53. Cash, D. M., Bocchetta, M., Thomas, D. L., Dick, K. M., van Swieten, J. C., Borroni, B., Galimberti, D., Masellis, M., Tartaglia, M. C., Rowe, J. B., Graff, C., Tagliavini, F., Frisoni, G. B., Laforce, R., Jr, Finger, E., de Mendonça, A., Sorbi, S., Rossor, M. N., Ourselin, S., Rohrer, J. D., … Genetic FTD Initiative, GENFI (2018). Patterns of gray matter atrophy in genetic frontotemporal dementia: results from the GENFI study. Neurobiology of aging, 62, 191–196.

54. Rohrer, J. D., Warren, J. D., Barnes, J., Mead, S., Beck, J., Pepple, T., Boyes, R., Omar, R., Collinge, J., Stevens, J. M., Warrington, E. K., Rossor, M. N., & Fox, N. C. (2008). Mapping the progression of progranulin-associated frontotemporal lobar degeneration. Nature clinical practice. Neurology, 4(8), 455–460.

55. Mahoney, C. J., Downey, L. E., Ridgway, G. R., Beck, J., Clegg, S., Blair, M., Finnegan, S., Leung, K. K., Yeatman, T., Golden, H., Mead, S., Rohrer, J. D., Fox, N. C., & Warren, J. D. (2012). Longitudinal neuroimaging and neuropsychological profiles of frontotemporal dementia with C9ORF72 expansions. Alzheimer's research & therapy, 4(5), 41.

56. Palmieri, A., Soraru, G., D'Ascenzo, C., Balestreri, S., Arcara, G., Ermani, M., Pegoraro, E., & Semenza, C. (2013). Specific numerical processing impairment in ALS patients. Amyotrophic lateral sclerosis & frontotemporal degeneration, 14(1), 6–12. https://doi.org/10.3109/17482968.2012.719235

57. Bartolomeo, P., Thiebaut de Schotten, M., & Chica, A. B. (2012). Brain networks of visuospatial attention and their disruption in visual neglect. Frontiers in human neuroscience, 6, 110.

For peer review only

58. Taipa, R., Tuna, A., Damásio, J., Pinto, P. S., Cavaco, S., Pereira, S., Milterberger-Miltenyi, G., Galimberti, D., & Melo-Pires, M. (2012). Clinical, neuropathological, and genetic characteristics of the novel IVS9+1delG GRN mutation in a patient with frontotemporal dementia. Journal of Alzheimer's disease : JAD, 30(1), 83–90.

59. Taghdiri, F., Sato, C., Ghani, M., Moreno, D., Rogaeva, E., & Tartaglia, M. C. (2016). Novel GRN Mutations in Patients with Corticobasal Syndrome. Scientific reports, 6, 22913.

60. Patel, A. N., & Sampson, J. B. (2015). Cognitive Profile of C9orf72 in Frontotemporal Dementia and Amyotrophic Lateral Sclerosis. Current neurology and neuroscience reports, 15(9), 59.

61. Benton, A. L., Hamsher, K., Varney, N. R., & Spreen, O. (1983). Contributions to neuropsychological assessment. New York: Oxford University Press.

62. Qualls, C. E., Bliwise, N. G., & Stringer, A. Y. (2000). Short forms of the Benton judgment of line orientation test: Development and psychometric properties. Archives of Clinical Neuropsychology, 15(2), 159–163.

63. Simard, M., van Reekum, R., & Myran, D. (2003). Visuospatial impairment in dementia with Lewy bodies and Alzheimer's disease: a process analysis approach. International journal of geriatric psychiatry, 18(5), 387–391.

64. Salimi, S., Irish, M., Foxe, D., Hodges, J. R., Piguet, O., & Burrell, J. R. (2017). Can visuospatial measures improve the diagnosis of Alzheimer's disease?. Alzheimer's & dementia (Amsterdam, Netherlands), 10, 66–74.

For peer review only

Figure Legends

Figure 1: Examples of tasks in the Ignite battery: top row left *Colour Mix*, top row middle *Swipe Out*, top row right *Card Sort*, middle row left *Path Finder*, middle row middle *Think Back*, middle row right *Balloon Fair*, bottom row left *Picture Pair*, bottom row middle *Sum Up*, bottom row right *Line Judge*.

Table 1: Summary of tasks within the Ignite protocol

	**Cognitive domain**	**Cognitive subdomain**	**Task**
1	Executive function and information processing speed	Inhibitory control	Colour Mix
**2**			Swipe Out
**3**		Cognitive flexibility	Card Sort
**4**			Path Finder
**5**		Working memory	Think Back
**6**		Decision making	Balloon Fair
**7**		Cognitive Timing	Time Tap
**8**	Social cognition	Emotion processing	Face Match
**9**		Theory of Mind	Mind Reading
**10**	Semantic knowledge		Picture Pair
**11**	Arithmetic		Sum Up
**12**	Visuospatial skills		Line Judge

For peer review only

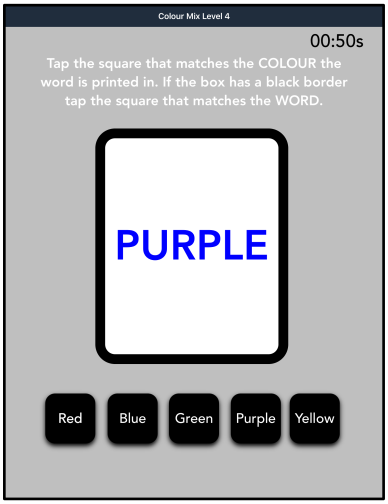
Colour Mix Level 4
00:50s
Tap the square that matches the COLOUR the word is printed in. If the box has a black border tap the square that matches the WORD.
PURPLE
Red
Blue
Green
Purple
Yellow

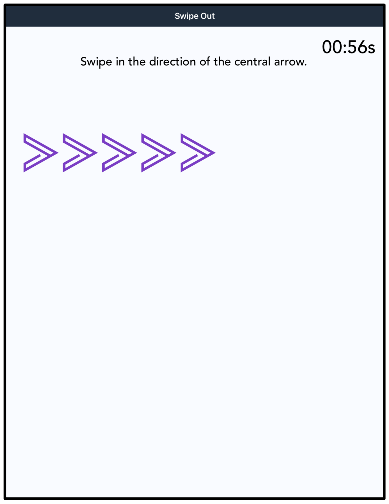
Swipe Out
00:56s
Swipe in the direction of the central arrow.

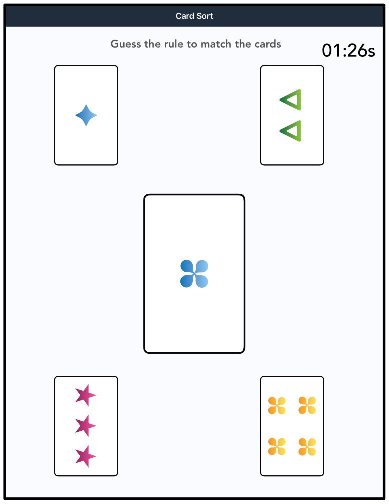
Card Sort
Guess the rule to match the cards
01:26s

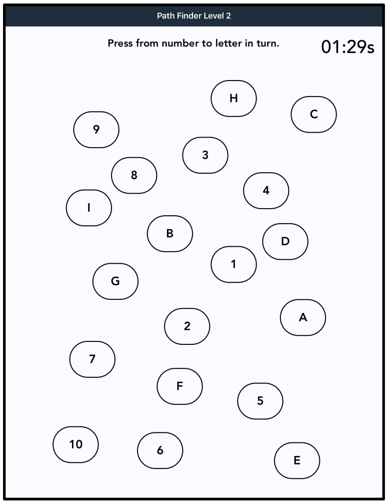
Path Finder Level 2
Press from number to letter in turn.
01:29s
H
C
9
3
8
4
I
B
D
1
G
A
2
7
F
5
10
6
E

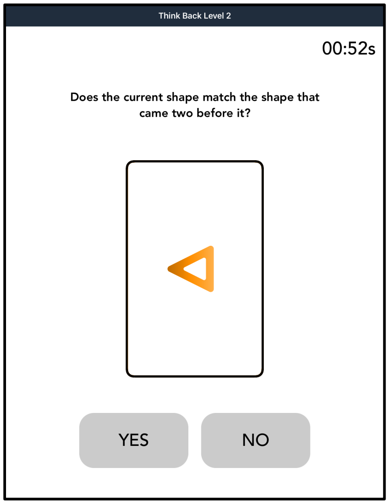
Think Back Level 2
00:52s
Does the current shape match the shape that came two before it?
YES
NO

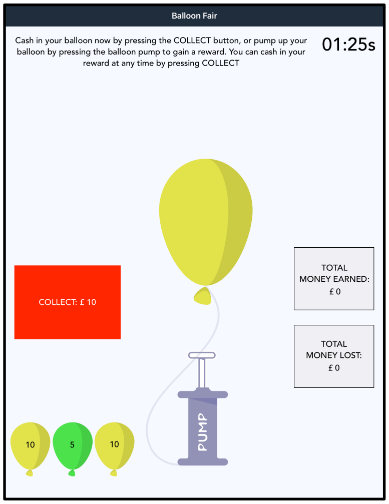
Balloon Fair
Cash in your balloon now by pressing the COLLECT button, or pump up your balloon by pressing the balloon pump to gain a reward. You can cash in your reward at any time by pressing COLLECT
01:25s
COLLECT: £ 10
TOTAL MONEY EARNED: £ 0
TOTAL MONEY LOST: £ 0
PUMP
10
5
10

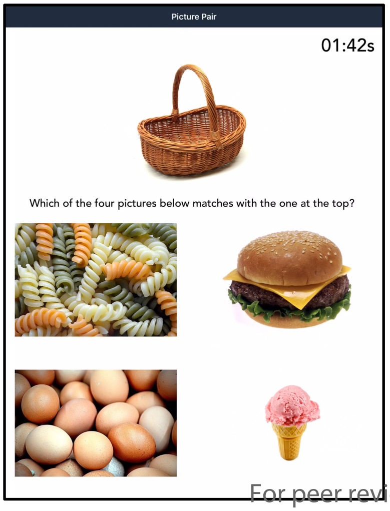
Picture Pair
01:42s
Which of the four pictures below matches with the one at the top?

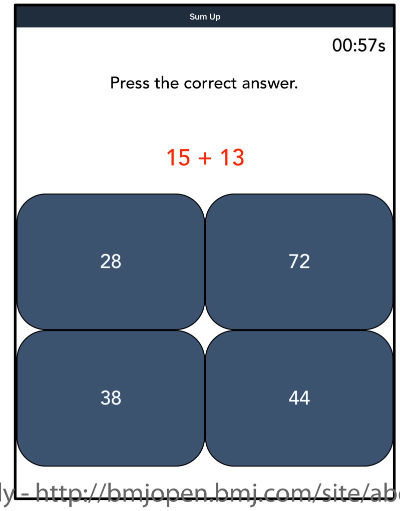
Sum Up
00:57s
Press the correct answer.
15 + 13
28
72
38
44

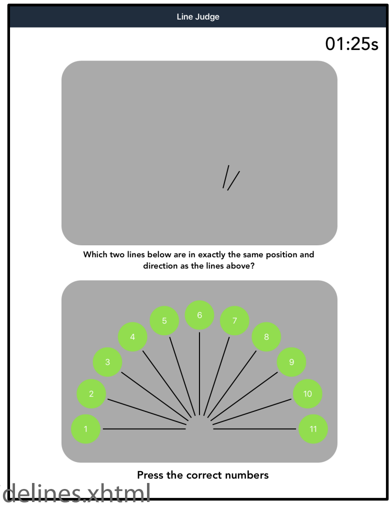
Line Judge
01:25s
Which two lines below are in exactly the same position and direction as the lines above?
1
2
3
4
5
6
7
8
9
10
11
Press the correct numbers

BMJ Open

Study protocol: computerized cognitive testing in a cohort of people with frontotemporal dementia

Journal:	*BMJ Open*
Manuscript ID	bmjopen-2021-055211.R3
Article Type:	Protocol
Date Submitted by the Author:	20-Jun-2022
Complete List of Authors:	Moore, Katrina; UCL Queen Square Institute of Neurology, Department of Neurodegenerative Disease<br>Convery, Rhian S.; UCL Queen Square Institute of Neurology, Department of Neurodegenerative Disease<br>Rohrer, Jonathan; UCL Queen Square Institute of Neurology, Department of Neurodegenerative Disease
**Primary Subject Heading**:	Neurology
Secondary Subject Heading:	Genetics and genomics
Keywords:	Dementia < NEUROLOGY, Adult neurology < NEUROLOGY, Delirium & cognitive disorders < PSYCHIATRY

SCHOLARONE™
Manuscripts

STUDY PROTOCOL

Main title

Study protocol: computerized cognitive testing in a cohort of people with frontotemporal dementia

Authors

Katrina M Moore PhD[1], Rhian S. Convery MSc[1], Jonathan D Rohrer FRCP PhD[1]

Affiliations

[1]Dementia Research Centre, Department of Neurodegenerative Disease, UCL Queen Square Institute of Neurology, London, UK.

Corresponding author

Professor Jonathan Rohrer, Dementia Research Centre, Department of Neurodegenerative Disease, UCL Institute of Neurology, Queen Square, London, WC1N 3BG, j.rohrer@ucl.ac.uk

For peer review only

Abstract

Introduction: The term frontotemporal dementia (FTD) refers to a heterogeneous group of neurodegenerative disorders affecting the frontal and temporal lobes. Cognitively, impairment of executive function and social cognition predominate across the FTD spectrum although other domains can be affected. Traditionally, cognition is tested through standard 'pen and paper' tasks in FTD. However, recent attempts have been made across other neurodegenerative disorders such as Alzheimer's disease to develop computerized batteries that allow more accurate and sensitive detection of cognitive impairment.

Methods and analysis: This paper describes the development of a novel battery of tests for a tablet computer, particularly focused on FTD. It consists of twelve different tasks which aim to tap into information processing speed, various aspects of executive function, social cognition, semantic knowledge, calculation and visuospatial skills. Future studies will focus on validating the battery in a healthy control cohort, comparing it against a standard 'pen and paper' psychometric battery, and finally testing it within an FTD cohort, including those with genetic forms of FTD where we will be able to assess its ability to detect very early cognitive deficits prior to the onset of symptoms.

Ethics and dissemination: Normative data will be produced in the initial validation study (approved by the UCL Ethics Committee, project ID 17691/002) and will be made available online.

Keywords: frontotemporal dementia, progranulin, tau, C9orf72, cognition

For peer review only

STRENGTHS AND LIMITATIONS

- This is one of the first computerized cognitive batteries to focus specifically on FTD.
- Tests are short and can be performed at home, reducing participant burden within studies.
- The study will require validation including comparison with a gold standard 'pen and paper' psychometric battery before it can be used more widely.

For peer review only

INTRODUCTION

Frontotemporal dementia (FTD) is a clinically, pathologically and genetically diverse neurodegenerative disease.[1] It is probably the commonest form of dementia in those under 60 years of age and is associated with two main clinical presentations: an impairment in personality and social conduct, known as behavioural variant FTD (bvFTD), and deficits in speech and language, called primary progressive aphasia (PPA). Around a third of FTD is familial,[2] with the majority of autosomal dominant FTD linked to mutations in three key genes: chromosome 9 open reading frame 72 (*C9orf72*), progranulin (*GRN*) and microtubule-associated protein tau (*MAPT*).

The cognitive deficits found in FTD are commonly in the executive function and social cognition domains for bvFTD,[3-5] and the language and semantic knowledge domains for those with PPA, [5-8] although there is often overlap between these disorders. Classically, episodic memory and more posterior cortical functions are spared, particularly in the early stages of the disease.[9] The trajectory of cognitive impairment has now been studied across a number of cohorts, with the earliest change being studied mostly within genetic cohorts where at-risk individuals who are many years from expected symptom onset can be investigated. Deficits across several cognitive tasks have been shown around five to eight years prior to symptom onset particularly in tests of executive function and social cognition, although the pattern of deficits can differ according to which mutation is carried.[10-16] However, prior studies have been performed mainly using traditional 'pen and paper' tasks which may well be limited in their ability to detect subtle changes. Using magnetic resonance imaging, neuroanatomical changes can be seen in presymptomatic genetic FTD studies much earlier, potentially over 20 years prior to expected symptom onset,[12] and so there is a need to bridge the gap between the sensitivity of neuroimaging techniques and current cognitive assessments.

For peer review only

Treatment trials in FTD are currently underway, and although many of the planned studies are focused on symptomatic FTD, it is likely that future studies will also include people in the presymptomatic phase of the disease. One of the greatest challenges facing trials of this nature is having robust cognitive outcome measures. The Food and Drug Administration have recognized that current assessments are not optimal, highlighting the need for improved cognitive markers as outcome measures for clinical trials,[17] and have encouraged the development of novel approaches to evaluate subtle deficits that may emerge in the presymptomatic stages of dementia.[18] Here we describe the design and protocol of Ignite, a novel computerized cognitive assessment tool designed for FTD. We outline the key objectives for developing a computerized battery and provide an overview of the cognitive domains tested within the battery.

METHODS AND ANALYSIS

Patient and Public Involvement

It is standard for traditional cognitive assessments to be performed by trained psychologists in hospital or research settings, which can be both time consuming and costly. On discussion with research participants they describe frequently experiencing high levels of anxiety in relation to attending research visits, particularly in relation to neuropsychological asssessment. In presymptomatic FTD studies this can be most prominent in those who have yet to find out their carrier status. In addition, the number of in person contact days required for a clinical trial can be burdensome and can prevent an individual's participation. With these considerations in mind, we wanted to design a computerized battery that could be performed by individuals themselves, with the aim of reducing participant anxiety, thus ensuring that their best performance is captured. We therefore initially discussed the development of this battery with a small group of people at-risk of familial FTD to ensure we could maximize the user-friendliness of the battery and reduce the participant burden.

For peer review only

Objectives for developing a computerized cognitive battery

Apart from improving the participant experience, we had a number of other objectives for developing the battery:

Comprehensiveness

We wanted to develop a set of assessments that could capture an individual's performance across a number of cognitive domains in a comprehensive manner for FTD. A major drawback of current cognitive assessments is the duration of testing required to get an accurate picture of individual performance, often taking a number of hours. The new battery was designed to be completed in around 30 minutes or less.

Increased sensitivity

Ignite consists mainly of tests which are modifications of existing cognitive tasks. However, each task was modified to shorten the time allowed for completion, with the measurement of reaction times aiming to increase the sensitivity to detect deficits.

Self-assessment

A key aim in the development of Ignite was its ability to be performed by individuals on their own, ideally while at home. All assessments were designed to be as intuitive as possible, appearing more like short games than standard cognitive tests, including the use of simple on-screen buttons or swipes to record answers. A challenge in designing tasks with self-assessment in mind is ensuring that participants are motivated to complete the tasks on their own and that they understand what is being asked of them. To assist participants in understanding and remembering task instructions, they are provided with brief written instructions prior to beginning each individual assessment task. Accompanying the written

For peer review only

instructions are short videos to ensure participants can clearly visualize and understand what is required of them. In addition, once a task has begun, a prompt of the task instruction is displayed at the top of the screen for the duration of the task. In this way, participants can refresh their memories of the task instructions at any time without having to exit the task. To keep participants motivated and engaged each task was designed to range from 30-180 seconds in duration, with most tasks programmed to time out after 60 seconds.

Protocol

Ignite consists of twelve separate assessments that measure a spectrum of cognitive functions (Table 1, Figure 1). Each test was included based on knowledge of the deficits seen in FTD, and in particular those domains most likely to be affected presymptomatically. The tests are self-administered in a predetermined order, in an environment familiar to the participant, such as their home. At present, the app is available on the iPad via the App Store, although future iterations will aim to make versions available for other tablets and operating systems.

Executive function

The term executive function describes a number of different aspects of cognition including reasoning, problem solving and planning [19] commonly related to the workings of the frontal lobe. It consists of a number of different subdomains, which include inhibitory control, cognitive flexibility and working memory.[20] We aimed to design tasks tapping into each of these subdomains as well as decision making and cognitive timing as previous studies of FTD have shown deficits within all of them,[21-23] and in particular impairment can be seen presymptomatically across each of the major genetic groups.[12,24]

Inhibitory control

For peer review only

This subdomain is described as the ability to control one's attention, behaviour and thoughts to override a strong internal predisposition or external lure.[20] It is abnormal in FTD and relates to the key symptom of disinhibition that forms part of the diagnostic criteria for behavioural variant FTD.[25] We included two tasks of inhibitory control.

Colour Mix

In our task, we adapted the classical Stroop task including five different colours: red, blue, green, purple and yellow. The initial two levels of the task test speed of processing. In Level 1, participants are presented with a coloured circle and all five colour names written below. They are asked to match the colour of the circle with the correct colour name from 5. They have 30 seconds to complete as many as possible (with a maximum of 50 possible trials). In Level 2, participants are presented with the name of a colour and (as with Level 1) the five colour names written below. They are asked to match the target colour word with the matching word below. They have 30 seconds to complete as many as possible (with a maximum of 50 possible trials). The next two levels measure inhibitory control. In Level 3, participants are presented with a colour word written in a different colour e.g. BLUE written in red ink. They are asked to match the colour of the ink (rather than the name) to one of the five colour names written below. They have 60 seconds to complete as many as possible (with a maximum of 50 possible trials). Lastly, in Level 4, participants are presented as in Level 3 with a colour word written in a different colour, and have to complete the task as in Level 3, unless a black border appears around the word (as it does on some trials), when they have to match the written word rather than the ink colour (e.g. match blue if the word is BLUE written in red ink) to one of the five colour names written below. They have 60 seconds to complete as many as possible (with a maximum of 50 possible trials).

Swipe Out

For peer review only

Our task was a modification of the classical Flanker task, with an arrowhead (facing up, down, left or right) with four flanking arrowheads surrounding the target arrowhead. Participants are asked to swipe in the direction of the central arrowhead. In some trials the flanking arrowheads are facing in the same direction, and in some trials they are facing in the opposite direction. Participants have 60 seconds to complete as many trials as possible (with a maximum of 40 possible trials).

Cognitive flexibility

This subdomain describes the ability to change perspectives from an established pattern, and mentally adjust or 'set shift' our perspective to new task demands. Tasks that tap into cognitive flexibility include the Wisconsin Card Sorting Task (WCST) [26] and the Trail Making Test (TMT) [27]. Deficits in both of these tasks have been previously described in FTD [10,13,28] including presymptomatically in those with genetic forms of FTD.[29]

Card Sort

Our task adapts a classical card sorting task, asking participants to sort cards by dragging them from the centre of the screen into card deposits located in the four corners of the screen. Participants are required to sort cards by a rule: either shape, number or colour. The primary aim of the task is for the participant to understand the correct sorting criteria on the basis of feedback, either correct or incorrect, and to switch flexibly between sorting rules whenever feedback indicates that the rule has changed. After a set number of correct card sorts are performed the rule changes, and negative feedback is given if participants continue to sort by the previous rule. The successful performance of the task is measured in participants' ability to flexibly respond to feedback given in shifting to the new rule.[26] The number of perseverative errors, identified by participants failing to adapt to a new rule, is thought to be indicative of poor cognitive

For peer review only

flexibility.[30] In our task, participants have a total of 90 seconds in order to sort as many as cards as possible.

Path Finder

This task adapts the Trail Making Test (TMT), asking participants to tap stimuli in sequential order (rather than draw an unbroken line between letters and/or numbers as in the original pen and paper task). Correct sequencing is indicated by the outline of each letter or number changing colour to green. The letter or number changes to red if selected incorrectly. In Level 1 participants are asked to select numbers in the correct order (similar to TMT part A), with a maximum of 60 seconds to complete the task. In Level 2 participants are asked to alternate between numbers and letters in the correct order (similar to TMT part B), with a maximum of 90 seconds to complete the task.

Working memory

This subdomain of executive function describes the ability to hold information in mind and mentally work with it.[31] A classic paradigm for assessing working memory is the N-back task, which requires participants to monitor a series of stimuli and respond whenever the stimuli presented matches the properties of the trial presented 'N' before it.[32] The task requires the monitoring, updating and manipulation of information displayed to perform the task effectively.

Think Back

There are two levels to our N-Back task. Level 1 is a 1-back task and Level 2 is a 2-back task. In Level 1 participants must indicate whether a shape matches the one preceding it. In Level 2 participants must indicate whether a shape matches the shape shown two before it, intended to place greater demand on

For peer review only

working memory. An answer is submitted by pressing a ‘yes’ or ‘no’ button. In each level, participants have 60 seconds to complete as many trials as possible (up to a maximum of 72 trials).

Decision making

For many people with FTD their ability to make decisions is compromised.[33] Several paradigms are employed in assessing decision making behaviour, often in the form of gambling or risk-taking tasks, such as the Iowa Gambling Task,[34] Cambridge Gambling Task,[35] Game of Dice Task [36] and the Balloon Analogue Risk Task,[37] with people with FTD shown to be impaired on these tests.[38]

Balloon Fair

In our task we adapted the Balloon Analogue Risk Task [37] to assess decision making behaviour. Participants are presented with a simulated balloon and balloon pump. Also presented is a button labelled ‘Collect £’ and a permanent money counter labelled ‘Total Earned’, alongside a counter displaying money that has been lost. With each press on the pump, the balloon is inflated, and money is accumulated in a temporary bank. Balloons pop after a predetermined ‘popping’ point, at which point the money accrued in the temporary bank is lost. Participants have the option to cash in the money accumulated in the temporary bank at any point by clicking the ‘Collect £’ button. After each balloon has popped (money lost) or the money has been collected a new balloon appears. Balloons are differently coloured (green, yellow and blue) with each colour associated with a unique sum (£5, £10, £50 respectively), and a unique popping point (quicker for higher sums). Participants are unaware of the probability of a balloon popping. The aim of the task is to earn as much money as possible. Participants have 90 seconds to complete the task.

Cognitive timing

For peer review only

The concept of timing is integral to performing many mental processes.[39] Although not well-studied in FTD, one previous study has shown impairment in both externally-paced and self-paced finger tapping.[23]

Time Tap

We adapted the task described in Henley et al., 2014 [23] to test cognitive timing. A pulsating circle is presented in the middle of the top of the screen and a tone sounds in time to the pulse (at 1500ms intervals). Participants are asked to use the index finger of their dominant hand to tap in time with the pulsating circle on a second circle presented below. After 30 seconds the pulsation and tone cease, and participants are instructed to maintain the same tempo for a further 30 seconds.

Social Cognition

Social cognition is the ability to perceive, interpret and generate a response to the intentions, behaviours, and feelings of others, and includes a number of subdomains such as emotion recognition and theory of mind. Social cognition impairment occurs early and almost universally in all people with FTD [40,41] as well as those with other forms of FTD, such as PPA.[42,43] These deficits include poor recognition of simple emotions in faces,[44,45] as well as impairment of complex emotion processing, and other tasks of theory of mind [45,46] Early deficits have been seen in genetic FTD including those who are presymptomatic.[13]

Face Match

In our task of simple emotion processing participants are presented with a target basic emotion word (sadness, happiness, anger, fear, disgust, surprise) at the top of the screen alongside nine images of faces in the corners of the screen.[47] Participants are asked to tap all of the faces displaying the target emotion as quickly as possible. There are five correct answers out of each nine presented faces, and participants

For peer review only

must press all five within 10 seconds, or the task moves on to the next set of nine faces. The participants have to complete as many items as possible within 60 seconds (up to a maximum of 6 sets of nine faces).

Mind Reading

Our task of complex emotion processing adapts the Reading the Mind in the Eyes test so that participants are presented with a target emotion word in the centre of the screen alongside four images of eyes in the corners of the screen, one of which matches the emotion. Participants are asked to select the image which best matches the target emotion word, completing as many as possible within 90 seconds (up to a maximum of 20 words).

Semantic knowledge

Impairment of semantic knowledge is characteristic of the semantic variant of primary progressive aphasia but is seen in other forms of FTD, and has been shown to occur presymptomatically, particularly in those with *MAPT* mutations.[48,49]

Picture Pair

Our task uses stimuli from the modified version of the Camel and Cactus task that we have previously described as detecting early semantic deficits in genetic FTD.[50] The participant has to match a target picture with a semantically-linked matching picture from a choice of four e.g. matching Camel with Cactus rather than a tree, sunflower or rose. The participants have to answer as many questions as possible in 120 seconds (with a maximum of 25 questions).

Arithmetic

For peer review only

Calculation abilities have been shown to be highly dependent on left parietal lobe function.[51,52] Although initial studies focused on anterior involvement of the frontal and temporal lobes in FTD, more recent studies have shown that some forms of FTD can have more posterior cortical atrophy, particularly those with *GRN* mutations and *C9orf72* expansions,[53] including presymptomatically.[12] Dyscalculia has been reported in both of these groups.[54,55] Although deficits are generally not limited to one form of mental arithmetic, one study has shown deficits specifically in multiplication in ALS.[56]

Sum Up

Our task includes additions, subtractions, multiplications and divisions, with participants presented with the sum and four possible answers, and a time limit of 10 seconds for completion of each sum. The participants have to answer as many questions as possible within 60 seconds (with a maximum of 48 sums, 12 of each type).

Visuospatial skills

Visuospatial processing is classically associated with right parietal lobe function.[57] As mentioned above, more posterior cortical atrophy, including involvement of the right parietal lobe has been seen in genetic FTD. Given this finding, it is unsurprising that several studies have highlighted impairments of visuospatial skills in both *GRN* mutation carriers [58,59] and *C9orf72* expansion carriers.[60]

Line Judge

Judgement of line orientation is a standard measure of visuospatial skills,[61,62] testing an individual's ability to match the angle and orientation of lines in space, with deficits seen in forms of dementia such as Alzheimer's diseases and Dementia with Lewy Bodies which are usually associated with parietal atrophy.[63,64]

For peer review only

In our task, participants are presented with pairs of angled lines and are asked to match the target pair of lines to a set of 11 lines arranged in a semicircle and numbered 1 to 11. Participants press the correct numbers that correspond to the line pair. The participants have to answer as many questions as possible within a 90 second timeframe (up to a maximum of 15 pairs).

Ethics and dissemination

Ignite is a novel computerized battery of assessments for the detection of cognitive changes in FTD. It includes tasks of information processing speed, executive function, social cognition, semantic knowledge, arithmetic and visuospatial skills. It potentially may be sensitive enough to detect early (and presymptomatic) deficits within an at-risk genetic cohort. Ignite will be initially validated in a healthy control cohort and compared against a standard set of 'pen and paper' psychometric tasks, including assessment of test-retest reliability. Normative data will be produced in this initial validation study (approved by the UCL Ethics Committee, project ID 17691/002) and will be made available online. The battery will then be tested within an FTD cohort such as GENFI (www.genfi.org; [12]) where it would be possible to assess both presymptomatic and symptomatic people with FTD. In the future we would also hope to be able to compare Ignite with other emerging computerized batteries aimed specifically at FTD, such as those being developed as part of the ARTFL-LEFFTDS Longitudinal Frontotemporal Lobar Degeneration (ALLFTD*: www.allftd.org)* study. The final goal of the Ignite study will be to develop a set of validated tasks which are able to track disease progression in FTD, specifically in the presymptomatic and very early symptomatic phase of the disease, and therefore have the potential to be employed in clinical trials as a cognitive outcome measure.

For peer review only

Abbreviations

FTD, frontotemporal dementia; PPA, primary progressive aphasia; C9orf72, chromosome 9 open reading frame 72; GRN, progranulin; MAPT, microtubule-associated protein tau; bvFTD, behavioural variant frontotemporal dementia, TMT, Trail Making Test; WCST, Wisconsin Card Sorting Task.

For peer review only

Declarations

Ethics approval and consent to participate

The study has been approved by the UCL ethics committee, project ID 17691/002. Written informed consent is being obtained for those taking part in the study.

Consent to publish

Not applicable

Availability of data and materials

The Ignite app is free to download and use from the Appstore.

Competing interests

KMM is now an employee of Ionis Pharmaceuticals. JDR has served on medical advisory boards or provided consultancy for Alector, Arkuda Therapeutics, Wave Life Sciences, Prevail Therapeutics, UCB, AC Immune, Astex Pharmaceuticals, Biogen, Takeda and Eisai.

Funding

The pilot version of the app was developed through an Alzheimer's Society PhD studentship (for KMM) awarded to JDR (AS-PhD-2015-005), with further development funded by a Frontotemporal Dementia Research Studentship in Memory of David Blechner awarded through The National Brain Appeal (RCN 290173) to RSC. JDR has received funding from an MRC Clinician Scientist Fellowship (MR/M008525/1), an NIHR Rare Disease Translational Research Collaboration (BRC149/NS/MH) and the Brain Research UK Miriam Marks Senior Fellowship; his work is also supported by the MRC UK GENFI grant (MR/M023664/1), the Bluefield Project and the JPND GENFI-PROX grant (2019-02248). None of the funding bodies had any direct input into the app development.

Authors' contributions

KMM, RSC and JDR wrote the manuscript. All authors have read and approved the manuscript.

Acknowledgements

We thank the research participants at the Dementia Research Centre for their contribution to the development of Ignite.

For peer review only

References

1. Warren, J. D., Rohrer, J. D., & Rossor, M. N. (2013). Clinical review. Frontotemporal dementia. BMJ (Clinical research ed.), 347, f4827.

2. Rohrer, J. D., Guerreiro, R., Vandrovcova, J., Uphill, J., Reiman, D., Beck, J., Isaacs, A. M., Authier, A., Ferrari, R., Fox, N. C., Mackenzie, I. R., Warren, J. D., de Silva, R., Holton, J., Revesz, T., Hardy, J., Mead, S., & Rossor, M. N. (2009). The heritability and genetics of frontotemporal lobar degeneration. Neurology, 73(18), 1451–1456.

3. Grossman M. (2002). Frontotemporal dementia: a review. Journal of the International Neuropsychological Society : JINS, 8(4), 566–583.

4. Harciarek, M., & Jodzio, K. (2005). Neuropsychological differences between frontotemporal dementia and Alzheimer's disease: a review. Neuropsychology review, 15(3), 131–145.

5. Harciarek, M., & Cosentino, S. (2013). Language, executive function and social cognition in the diagnosis of frontotemporal dementia syndromes. International review of psychiatry (Abingdon, England), 25(2), 178–196.

6. Warren, J. D., Rohrer, J. D., & Rossor, M. N. (2013). Clinical review. Frontotemporal dementia. BMJ (Clinical research ed.), 347, f4827.

7. Woollacott, I. O., & Rohrer, J. D. (2016). The clinical spectrum of sporadic and familial forms of frontotemporal dementia. Journal of neurochemistry, 138 Suppl 1, 6–31.

8. Deleon, J., & Miller, B. L. (2018). Frontotemporal dementia. Handbook of clinical neurology, 148, 409–430.

9. Gorno-Tempini, M. L., Hillis, A. E., Weintraub, S., Kertesz, A., Mendez, M., Cappa, S. F., Ogar, J. M., Rohrer, J. D., Black, S., Boeve, B. F., Manes, F., Dronkers, N. F., Vandenberghe, R., Rascovsky, K., Patterson, K., Miller, B. L., Knopman, D. S., Hodges, J. R., Mesulam, M. M., & Grossman, M.

For peer review only

(2011). Classification of primary progressive aphasia and its variants. Neurology, 76(11), 1006–1014.

10. Geschwind, D. H., Robidoux, J., Alarcón, M., Miller, B. L., Wilhelmsen, K. C., Cummings, J. L., & Nasreddine, Z. S. (2001). Dementia and neurodevelopmental predisposition: cognitive dysfunction in presymptomatic subjects precedes dementia by decades in frontotemporal dementia. Annals of neurology, 50(6), 741–746.

11. Hallam, B. J., Jacova, C., Hsiung, G. Y., Wittenberg, D., Sengdy, P., Bouchard-Kerr, P., Slack, P., Rademakers, R., Baker, M., Chow, T. W., Levine, B., Feldman, H. H., & Mackenzie, I. R. (2014). Early neuropsychological characteristics of progranulin mutation carriers. Journal of the International Neuropsychological Society : JINS, 20(7), 694–703.

12. Rohrer, J. D., Nicholas, J. M., Cash, D. M., van Swieten, J., Dopper, E., Jiskoot, L., van Minkelen, R., Rombouts, S. A., Cardoso, M. J., Clegg, S., Espak, M., Mead, S., Thomas, D. L., De Vita, E., Masellis, M., Black, S. E., Freedman, M., Keren, R., MacIntosh, B. J., Rogaeva, E., … Binetti, G. (2015). Presymptomatic cognitive and neuroanatomical changes in genetic frontotemporal dementia in the Genetic Frontotemporal dementia Initiative (GENFI) study: a cross-sectional analysis. The Lancet. Neurology, 14(3), 253–262.

13. Jiskoot, L. C., Dopper, E. G., Heijer, T. d., Timman, R., van Minkelen, R., van Swieten, J. C., & Papma, J. M. (2016). Presymptomatic cognitive decline in familial frontotemporal dementia: A longitudinal study. Neurology, 87(4), 384–391.

14. Papma, J. M., Jiskoot, L. C., Panman, J. L., Dopper, E. G., den Heijer, T., Donker Kaat, L., Pijnenburg, Y., Meeter, L. H., van Minkelen, R., Rombouts, S., & van Swieten, J. C. (2017). Cognition and gray and white matter characteristics of presymptomatic C9orf72 repeat expansion. Neurology, 89(12), 1256–1264.

For peer review only

15. Suhonen, N. M., Haanpää, R. M., Korhonen, V., Jokelainen, J., Pitkäniemi, A., Heikkinen, A. L., Krüger, J., Hartikainen, P., Helisalmi, S., Hiltunen, M., Hänninen, T., & Remes, A. M. (2017). Neuropsychological Profile in the C9ORF72 Associated Behavioral Variant Frontotemporal Dementia. Journal of Alzheimer's disease : JAD, 58(2), 479–489.

16. Jiskoot, L. C., Panman, J. L., van Asseldonk, L., Franzen, S., Meeter, L., Donker Kaat, L., van der Ende, E. L., Dopper, E., Timman, R., van Minkelen, R., van Swieten, J. C., van den Berg, E., & Papma, J. M. (2018). Longitudinal cognitive biomarkers predicting symptom onset in presymptomatic frontotemporal dementia. Journal of neurology, 265(6), 1381–1392.

17. Sabbagh, M. N., Hendrix, S., & Harrison, J. E. (2019). FDA position statement "Early Alzheimer's disease: Developing drugs for treatment, Guidance for Industry". Alzheimer's & dementia (New York, N. Y.), 5, 13–19.

18. FDA (2018). Early Alzheimer's Disease: Developing Drugs for Treatment Guidance for Industry DRAFT GUIDANCE. Retrieved from https://www.fda.gov/Drugs/GuidanceComplianceRegulatoryInformation/Guidances/default.htm

19. Collins, A., & Koechlin, E. (2012). Reasoning, learning, and creativity: frontal lobe function and human decision-making. PLoS biology, 10(3), e1001293.

20. Diamond A. (2013). Executive functions. Annual review of psychology, 64, 135–168.

21. Strenziok, M., Pulaski, S., Krueger, F., Zamboni, G., Clawson, D., & Grafman, J. (2011). Regional brain atrophy and impaired decision making on the balloon analog risk task in behavioral variant frontotemporal dementia. Cognitive and behavioral neurology : official journal of the Society for Behavioral and Cognitive Neurology, 24(2), 59–67.

22. Manes, F., Torralva, T., Ibáñez, A., Roca, M., Bekinschtein, T., & Gleichgerrcht, E. (2011). Decision-making in frontotemporal dementia: clinical, theoretical and legal implications. Dementia and geriatric cognitive disorders, 32(1), 11–17.

For peer review only

23. Henley, S. M., Downey, L. E., Nicholas, J. M., Kinnunen, K. M., Golden, H. L., Buckley, A., Mahoney, C. J., & Crutch, S. J. (2014). Degradation of cognitive timing mechanisms in behavioural variant frontotemporal dementia. Neuropsychologia, 65, 88–101.

24. Staffaroni, A. M., Bajorek, L., Casaletto, K. B., Cobigo, Y., Goh, S. M., Wolf, A., Heuer, H. W., Elahi, F. M., Ljubenkov, P. A., Dever, R., Kornak, J., Appleby, B., Bove, J., Bordelon, Y., Brannelly, P., Brushaber, D., Caso, C., Coppola, G., Dheel, C., Dickerson, B. C., … ARTFL/LEFFTDS consortium (2020). Assessment of executive function declines in presymptomatic and mildly symptomatic familial frontotemporal dementia: NIH-EXAMINER as a potential clinical trial endpoint. Alzheimer's & dementia : the journal of the Alzheimer's Association, 16(1), 11–21.

25. Rascovsky, K., Hodges, J. R., Knopman, D., Mendez, M. F., Kramer, J. H., Neuhaus, J., van Swieten, J. C., Seelaar, H., Dopper, E. G., Onyike, C. U., Hillis, A. E., Josephs, K. A., Boeve, B. F., Kertesz, A., Seeley, W. W., Rankin, K. P., Johnson, J. K., Gorno-Tempini, M. L., Rosen, H., Prioleau-Latham, C. E., … Miller, B. L. (2011). Sensitivity of revised diagnostic criteria for the behavioural variant of frontotemporal dementia. Brain : a journal of neurology, 134(Pt 9), 2456–2477.

26. Stuss, D. T., Levine, B., Alexander, M. P., Hong, J., Palumbo, C., Hamer, L., Murphy, K. J., & Izukawa, D. (2000). Wisconsin Card Sorting Test performance in patients with focal frontal and posterior brain damage: effects of lesion location and test structure on separable cognitive processes. Neuropsychologia, 38(4), 388–402.

27. Delis, D. ., Kaplan, E., & J.H, K. (2001). The Delis-Kaplan Executive Function System: Examiner's Manual. San Andonio: The Psychological Corporation.

28. Rohrer, J. D., Warren, J. D., Omar, R., Mead, S., Beck, J., Revesz, T., Holton, J., Stevens, J. M., Al-Sarraj, S., Pickering-Brown, S. M., Hardy, J., Fox, N. C., Collinge, J., Warrington, E. K., & Rossor, M. N. (2008). Parietal lobe deficits in frontotemporal lobar degeneration caused by a mutation in the progranulin gene. Archives of neurology, 65(4), 506–513.

For peer review only

29. Barandiaran, M., Estanga, A., Moreno, F., Indakoetxea, B., Alzualde, A., Balluerka, N., Martí Massó, J. F., & López de Munain, A. (2012). Neuropsychological features of asymptomatic c.709-1G>A progranulin mutation carriers. Journal of the International Neuropsychological Society : JINS, 18(6), 1086–1090.

30. Lange, F., Brückner, C., Knebel, A., Seer, C., & Kopp, B. (2018). Executive dysfunction in Parkinson's disease: A meta-analysis on the Wisconsin Card Sorting Test literature. Neuroscience and biobehavioral reviews, 93, 38–56.

31. Baddeley A.D, & Hitch G.J. (1994). Developments in the Concept of Working Memory. Neuropsychology, 8, 485–493.

32. Owen, A. M., McMillan, K. M., Laird, A. R., & Bullmore, E. (2005). N-back working memory paradigm: a meta-analysis of normative functional neuroimaging studies. Human brain mapping, 25(1), 46–59.

33. Gleichgerrcht, E., Torralva, T., Roca, M., Szenkman, D., Ibanez, A., Richly, P., Pose, M., & Manes, F. (2012). Decision making cognition in primary progressive aphasia. Behavioural neurology, 25(1), 45–52.

34. Bechara, A., & Van Der Linden, M. (2005). Decision-making and impulse control after frontal lobe injuries. Current opinion in neurology, 18(6), 734–739.

35. Rogers, R. D., Owen, A. M., Middleton, H. C., Williams, E. J., Pickard, J. D., Sahakian, B. J., & Robbins, T. W. (1999). Choosing between small, likely rewards and large, unlikely rewards activates inferior and orbital prefrontal cortex. The Journal of neuroscience : the official journal of the Society for Neuroscience, 19(20), 9029–9038.

36. Brand, M., Fujiwara, E., Borsutzky, S., Kalbe, E., Kessler, J., & Markowitsch, H. J. (2005). Decision-making deficits of korsakoff patients in a new gambling task with explicit rules: associations with executive functions. Neuropsychology, 19(3), 267–277.

For peer review only

37. Lejuez, C. W., Read, J. P., Kahler, C. W., Richards, J. B., Ramsey, S. E., Stuart, G. L., Strong, D. R., & Brown, R. A. (2002). Evaluation of a behavioral measure of risk taking: the Balloon Analogue Risk Task (BART). Journal of experimental psychology. Applied, 8(2), 75–84.

38. Rahman, S., Sahakian, B. J., Hodges, J. R., Rogers, R. D., & Robbins, T. W. (1999). Specific cognitive deficits in mild frontal variant frontotemporal dementia. Brain : a journal of neurology, 122 (Pt 8), 1469–1493.

39. Allman, M. J., & Meck, W. H. (2012). Pathophysiological distortions in time perception and timed performance. Brain : a journal of neurology, 135(Pt 3), 656–677.

40. Snowden, J. S., Gibbons, Z. C., Blackshaw, A., Doubleday, E., Thompson, J., Craufurd, D., Foster, J., Happé, F., & Neary, D. (2003). Social cognition in frontotemporal dementia and Huntington's disease. Neuropsychologia, 41(6), 688–701.

41. Russell, L. L., Greaves, C. V., Bocchetta, M., Nicholas, J., Convery, R. S., Moore, K., Cash, D. M., van Swieten, J., Jiskoot, L., Moreno, F., Sanchez-Valle, R., Borroni, B., Laforce, R., Jr, Masellis, M., Tartaglia, M. C., Graff, C., Rotondo, E., Galimberti, D., Rowe, J. B., Finger, E., … Genetic FTD Initiative, GENFI (2020). Social cognition impairment in genetic frontotemporal dementia within the GENFI cohort. Cortex; a journal devoted to the study of the nervous system and behavior, 133, 384–398.

42. Couto, B., Manes, F., Montañés, P., Matallana, D., Reyes, P., Velasquez, M., Yoris, A., Baez, S., & Ibáñez, A. (2013). Structural neuroimaging of social cognition in progressive non-fluent aphasia and behavioral variant of frontotemporal dementia. Frontiers in human neuroscience, 7, 467.

43. Fittipaldi, S., Ibanez, A., Baez, S., Manes, F., Sedeno, L., & Garcia, A. M. (2019). More than words: Social cognition across variants of primary progressive aphasia. Neuroscience and biobehavioral reviews, 100, 263–284.

For peer review only

44. Kumfor, F., & Piguet, O. (2012). Disturbance of emotion processing in frontotemporal dementia: a synthesis of cognitive and neuroimaging findings. Neuropsychology review, 22(3), 280–297.

45. Lough, S., Kipps, C. M., Treise, C., Watson, P., Blair, J. R., & Hodges, J. R. (2006). Social reasoning, emotion and empathy in frontotemporal dementia. Neuropsychologia, 44(6), 950–958.

46. Gregory, C., Lough, S., Stone, V., Erzinclioglu, S., Martin, L., Baron-Cohen, S., & Hodges, J. R. (2002). Theory of mind in patients with frontal variant frontotemporal dementia and Alzheimer's disease: theoretical and practical implications. Brain : a journal of neurology, 125(Pt 4), 752–764.

47. Tottenham, N., Tanaka, J. W., Leon, A. C., McCarry, T., Nurse, M., Hare, T. A., Marcus, D. J., Westerlund, A., Casey, B. J., & Nelson, C. (2009). The NimStim set of facial expressions: judgments from untrained research participants. Psychiatry research, 168(3), 242–249.

48. Snowden, J. S., Adams, J., Harris, J., Thompson, J. C., Rollinson, S., Richardson, A., Jones, M., Neary, D., Mann, D. M., & Pickering-Brown, S. (2015). Distinct clinical and pathological phenotypes in frontotemporal dementia associated with MAPT, PGRN and C9orf72 mutations. Amyotrophic lateral sclerosis & frontotemporal degeneration, 16(7-8), 497–505.

49. Hardy, C. J., Buckley, A. H., Downey, L. E., Lehmann, M., Zimmerer, V. C., Varley, R. A., Crutch, S. J., Rohrer, J. D., Warrington, E. K., & Warren, J. D. (2016). The Language Profile of Behavioral Variant Frontotemporal Dementia. Journal of Alzheimer's disease : JAD, 50(2), 359–371.

50. Moore, K., Convery, R., Bocchetta, M., Neason, M., Cash, D. M., Greaves, C., Russell, L. L., Clarke, M., Peakman, G., van Swieten, J., Jiskoot, L., Moreno, F., Barandiaran, M., Sanchez-Valle, R., Borroni, B., Laforce, R., Jr, Doré, M. C., Masellis, M., Tartaglia, M. C., Graff, C., … Genetic FTD Initiative, GENFI* (2020). A modified Camel and Cactus Test detects presymptomatic semantic impairment in genetic frontotemporal dementia within the GENFI cohort. Applied neuropsychology. Adult, 1–8.

For peer review only

51. Takayama, Y., Sugishita, M., Akiguchi, I., & Kimura, J. (1994). Isolated acalculia due to left parietal lesion. Archives of neurology, 51(3), 286–291.

52. Dehaene, S., Piazza, M., Pinel, P., & Cohen, L. (2003). Three parietal circuits for number processing. Cognitive neuropsychology, 20(3), 487–506.

53. Cash, D. M., Bocchetta, M., Thomas, D. L., Dick, K. M., van Swieten, J. C., Borroni, B., Galimberti, D., Masellis, M., Tartaglia, M. C., Rowe, J. B., Graff, C., Tagliavini, F., Frisoni, G. B., Laforce, R., Jr, Finger, E., de Mendonça, A., Sorbi, S., Rossor, M. N., Ourselin, S., Rohrer, J. D., … Genetic FTD Initiative, GENFI (2018). Patterns of gray matter atrophy in genetic frontotemporal dementia: results from the GENFI study. Neurobiology of aging, 62, 191–196.

54. Rohrer, J. D., Warren, J. D., Barnes, J., Mead, S., Beck, J., Pepple, T., Boyes, R., Omar, R., Collinge, J., Stevens, J. M., Warrington, E. K., Rossor, M. N., & Fox, N. C. (2008). Mapping the progression of progranulin-associated frontotemporal lobar degeneration. Nature clinical practice. Neurology, 4(8), 455–460.

55. Mahoney, C. J., Downey, L. E., Ridgway, G. R., Beck, J., Clegg, S., Blair, M., Finnegan, S., Leung, K. K., Yeatman, T., Golden, H., Mead, S., Rohrer, J. D., Fox, N. C., & Warren, J. D. (2012). Longitudinal neuroimaging and neuropsychological profiles of frontotemporal dementia with C9ORF72 expansions. Alzheimer's research & therapy, 4(5), 41.

56. Palmieri, A., Soraràu, G., D'Ascenzo, C., Balestreri, S., Arcara, G., Ermani, M., Pegoraro, E., & Semenza, C. (2013). Specific numerical processing impairment in ALS patients. Amyotrophic lateral sclerosis & frontotemporal degeneration, 14(1), 6–12. https://doi.org/10.3109/17482968.2012.719235

57. Bartolomeo, P., Thiebaut de Schotten, M., & Chica, A. B. (2012). Brain networks of visuospatial attention and their disruption in visual neglect. Frontiers in human neuroscience, 6, 110.

For peer review only

58. Taipa, R., Tuna, A., Damásio, J., Pinto, P. S., Cavaco, S., Pereira, S., Milterberger-Miltenyi, G., Galimberti, D., & Melo-Pires, M. (2012). Clinical, neuropathological, and genetic characteristics of the novel IVS9+1delG GRN mutation in a patient with frontotemporal dementia. Journal of Alzheimer's disease : JAD, 30(1), 83–90.

59. Taghdiri, F., Sato, C., Ghani, M., Moreno, D., Rogaeva, E., & Tartaglia, M. C. (2016). Novel GRN Mutations in Patients with Corticobasal Syndrome. Scientific reports, 6, 22913.

60. Patel, A. N., & Sampson, J. B. (2015). Cognitive Profile of C9orf72 in Frontotemporal Dementia and Amyotrophic Lateral Sclerosis. Current neurology and neuroscience reports, 15(9), 59.

61. Benton, A. L., Hamsher, K., Varney, N. R., & Spreen, O. (1983). Contributions to neuropsychological assessment. New York: Oxford University Press.

62. Qualls, C. E., Bliwise, N. G., & Stringer, A. Y. (2000). Short forms of the Benton judgment of line orientation test: Development and psychometric properties. Archives of Clinical Neuropsychology, 15(2), 159–163.

63. Simard, M., van Reekum, R., & Myran, D. (2003). Visuospatial impairment in dementia with Lewy bodies and Alzheimer's disease: a process analysis approach. International journal of geriatric psychiatry, 18(5), 387–391.

64. Salimi, S., Irish, M., Foxe, D., Hodges, J. R., Piguet, O., & Burrell, J. R. (2017). Can visuospatial measures improve the diagnosis of Alzheimer's disease?. Alzheimer's & dementia (Amsterdam, Netherlands), 10, 66–74.

For peer review only

Figure Legends

Figure 1: Examples of tasks in the Ignite battery: top row left *Colour Mix*, top row middle *Swipe Out*, top row right *Card Sort*, middle row left *Path Finder*, middle row middle *Think Back*, middle row right *Balloon Fair*, bottom row left *Picture Pair*, bottom row middle *Sum Up*, bottom row right *Line Judge*.

Table 1: Summary of tasks within the Ignite protocol

	**Cognitive domain**	**Cognitive subdomain**	**Task**
1	Executive function and information processing speed	Inhibitory control	Colour Mix
**2**			Swipe Out
**3**		Cognitive flexibility	Card Sort
**4**			Path Finder
**5**		Working memory	Think Back
**6**		Decision making	Balloon Fair
**7**		Cognitive Timing	Time Tap
**8**	Social cognition	Emotion processing	Face Match
**9**		Theory of Mind	Mind Reading
**10**	Semantic knowledge		Picture Pair
**11**	Arithmetic		Sum Up
**12**	Visuospatial skills		Line Judge

For peer review only

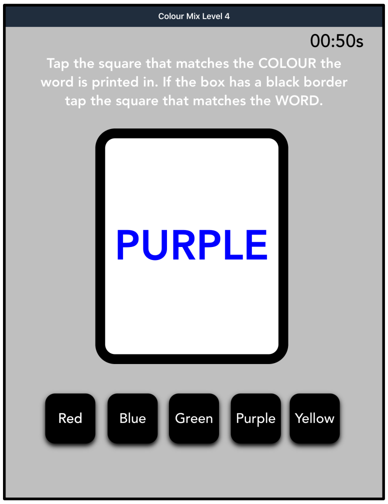
Colour Mix Level 4
00:50s
Tap the square that matches the COLOUR the word is printed in. If the box has a black border tap the square that matches the WORD.
PURPLE
Red
Blue
Green
Purple
Yellow

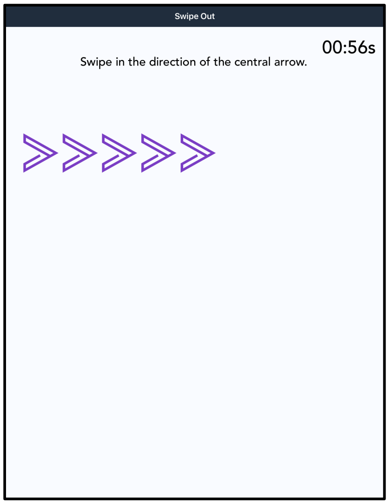
Swipe Out
00:56s
Swipe in the direction of the central arrow.

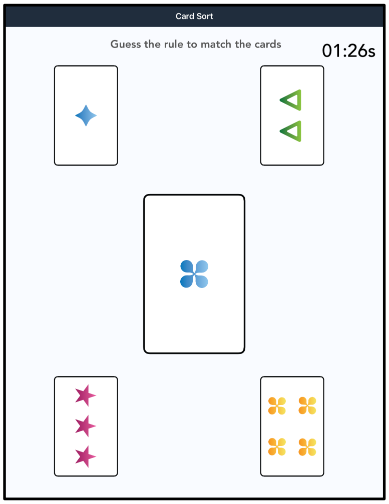
Card Sort
Guess the rule to match the cards
01:26s

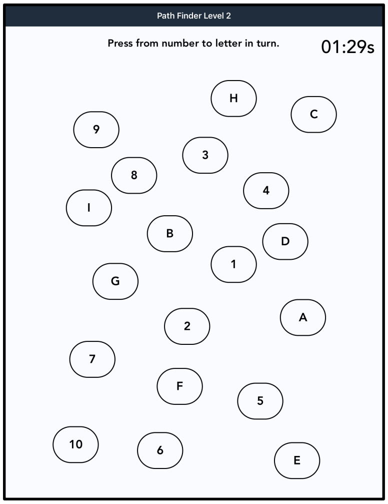
Path Finder Level 2
Press from number to letter in turn.
01:29s
H
C
9
3
8
4
I
B
D
1
G
A
2
7
F
5
10
6
E

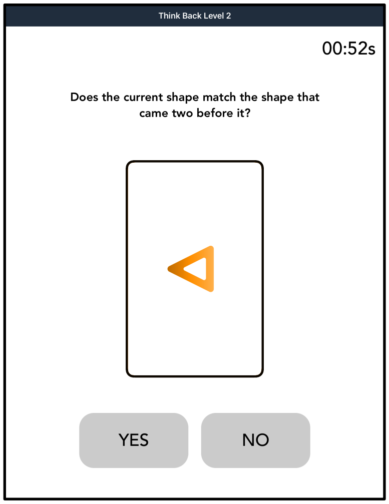
Think Back Level 2
00:52s
Does the current shape match the shape that came two before it?
YES
NO

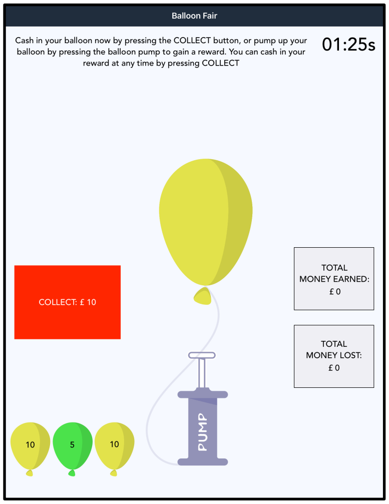
Balloon Fair
Cash in your balloon now by pressing the COLLECT button, or pump up your balloon by pressing the balloon pump to gain a reward. You can cash in your reward at any time by pressing COLLECT
01:25s
TOTAL MONEY EARNED: £ 0
COLLECT: £ 10
TOTAL MONEY LOST: £ 0
PUMP
10
5
10

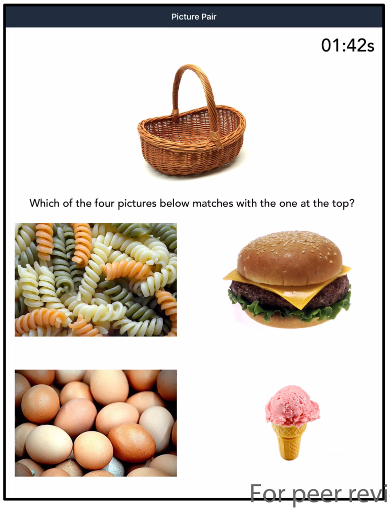
Picture Pair
01:42s
Which of the four pictures below matches with the one at the top?

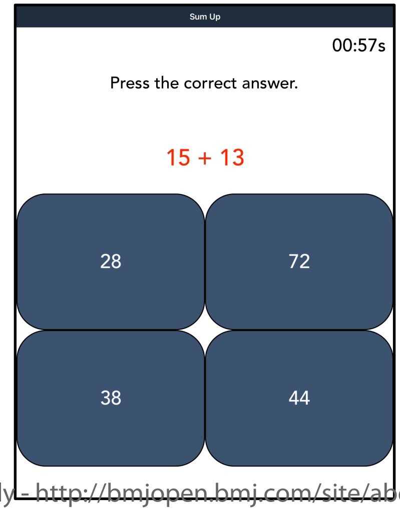
Sum Up
00:57s
Press the correct answer.
15 + 13
28
72
38
44

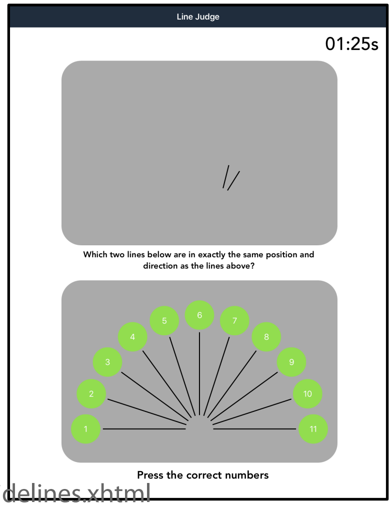
Line Judge
01:25s
Which two lines below are in exactly the same position and direction as the lines above?
1
2
3
4
5
6
7
8
9
10
11
Press the correct numbers
